# MicroRNA-145 attenuates TNF-*α*-driven cartilage matrix degradation in osteoarthritis via direct suppression of MKK4

**DOI:** 10.1038/cddis.2017.522

**Published:** 2017-10-26

**Authors:** Guoli Hu, Xiaoying Zhao, Chuandong Wang, Yiyun Geng, Jingyu Zhao, Jiajia Xu, Bin Zuo, Chen Zhao, Chenglong Wang, Xiaoling Zhang

**Affiliations:** 1 Department of Orthopedic Surgery, Xin Hua Hospital Affiliated to Shanghai Jiao Tong University School of Medicine (SJTUSM), Shanghai 200092, China; 2 The Key Laboratory of Stem Cell Biology, Institute of Health Sciences, Shanghai Jiao Tong University School of Medicine (SJTUSM) & Shanghai Institutes for Biological Sciences (SIBS), Chinese Academy of Sciences (CAS), Shanghai 200031, China; 3 Department of Orthopedic Surgery, Rui Jin Hospital Affiliated to Shanghai Jiao Tong University School of Medicine (SJTUSM), Shanghai 200025, China

## Abstract

Cartilage dyshomeostasis contributes to osteoarthritis (OA) pathogenesis, and tumor necrosis factor (TNF)-*α* has critical role in this process by driving inflammatory cascades and cartilage degradation. However, the negative regulation of TNF-*α*-mediated signaling remains undefined. Here we demonstrate the crucial role of miR-145 in the modulation of TNF-*α*-mediated signaling and cartilage matrix degradation. MicroRNA (miRNA) expression profiles of TNF-*α*-stimulated chondrocytes showed that miR-145 expression was rapidly downregulated by TNF-*α*. Moreover, miR-145 was directly repressed by p65 and was negatively correlated with TNF-*α* secretion during OA progression. Further, we found that miR-145 directly targeted mitogen-activated protein kinase kinase 4 (MKK4) and broadly restrained the production of several TNF-*α*-triggered matrix-degrading enzymes (MMP-3, MMP-13, and Adamts-5). Mechanistic studies unveiled that miR-145 negatively regulated TNF-*α*-mediated JNK and p38 activation, as well as the nuclear accumulation of p-c-Jun and p-ATF2, by inhibiting MKK4 phosphorylation, eventually resulting in the alteration of catabolic genes transcription. Indeed, p-ATF2 interacted with the promoter of *Mmp-13*, whereas p-c-Jun bound to promoters of *Mmp-3* and *Adamts-5*. MKK4 was significantly elevated in OA cartilage. Eliminating MKK4 by short hairpin RNA resulted in obviously decreased matrix-degrading enzymes production, JNK and p38 inactivation, and an inhibition of cartilage degradation. On the contrary, MKK4 overexpression enhanced TNF-*α*-mediated signaling activation and transcription of downstream catabolic genes, and consequently worsened cartilage degradation. Moreover, intra-articular (IA) injection of miR-145 agonist to rat with surgery-induced OA alleviated cartilage destruction. Altogether, we elucidate a novel regulatory mechanism underlying TNF-*α*-triggered cartilage degradation and demonstrate the potential utility of miR-145 and MKK4 as therapy targets for OA.

Osteoarthritis (OA) is the most prevalent chronic joint disease characterized by a group of abnormalities, such as cartilage destruction, subchondral bone remodeling, and synovial inflammation.^1^ These conditions have been mainly attributed to an imbalance between the anabolism and catabolism of the articular cartilage, especially an increase in catabolism.^2^ Proinflammatory cytokines, such as interleukin-1*β* (IL-1*β*), tumor necrosis factor (TNF-*α*), and IL-6, are critical mediators in this dyshomeostasis.^3, 4, 5^ In general, cytokines such as IL-1*β* and TNF-*α* increase matrix-degrading enzyme (matrix metalloproteinases, MMPs and a disintegrin and metalloproteinase with thrombospondin motifs, ADAMTS) gene expression, which in turn degrade the cartilage extracellular matrix (ECM), and then the degraded ECM fragments further facilitate synovial inflammation and the production of proinflammatory cytokines, and eventually lead to cartilage destruction.^4, 6^ On the other hand, cytokines also blunt the synthesis of the cartilage ECM.^7^ Therefore, molecules possessing anti-inflammatory properties are urgently needed.

It is known that inflammation presents cascade amplification,^8^ implying that signaling cascades consisting of protein kinases, especially the upstream molecules involved in NF-*κ*B and mitogen-activated protein kinases (MAPK) signaling pathways, may have vital roles in driving the inflammatory cascade during OA. In addition, as the main cytokines involved in OA pathogenesis, IL-1*β* is mainly associated with cartilage destruction, whereas TNF-*α* is implicated in driving of the inflammatory cascades during OA.^4, 9^ Furthermore, chondrocytes from human OA cartilage present abnormal high expression of the p55 TNF-*α* receptor, which renders OA cartilage, particularly susceptible to TNF-*α* degradative stimuli. Meanwhile, OA cartilage produces more TNF-*α* and TNF angle convertase enzyme compared with normal cartilage.^10^ Therefore, targeting TNF-*α* signaling may be a powerful strategy to cure OA.

Mechanisms that mediate the actions of TNF-*α* have been intensively studied.^11, 12^ In general, upon TNF-*α* binding to TNF receptor (TNFR), a conformational change promotes recruitment of cytosolic factors to the TNFR intracellular death domain (DD). Subsequently, TNFR-associated DD (TRADD) is recruited directly via a homotypic DD–DD interaction, and then the TRADD adaptor recruits a DD-containing kinase termed receptor interacting protein-1 (RIP1). RIP1 in turn recruits TNF receptor associated factor-2 (TRAF2) and/or TRAF5, as well as cellular inhibitor of apoptosis protein-1 (cIAP1) and cIAP2. These adaptors act in concert with RIP1 to engage the downstream phosphorylation cascades to activate NF-*κ*B, JNK, and p38 pathways, which ultimately promote inflammation and transcriptional activation.^13, 14, 15^ However, the negative regulation of TNF-*α*-mediated signal transduction, especially the modulation of upstream molecules still remains largely unknown.

MicroRNAs (miRNAs) are small noncoding RNAs of 19–24 nucleotides in length and exert their regulatory functions by mRNA degradation or translational inhibition.^16, 17, 18, 19^ MiRNAs regulate ~30% of human protein-coding genes, implying the essential role of miRNAs in controlling gene expression.^20, 21, 22^ Recent studies have illuminated several miRNAs related to cartilage breakdown triggered by IL-1*β*, including miR-140,^23^ miR-27b,^24^ miR-146a,^25, 26^ miR-98,^27^ miR-558,^28^ and miR-30a,^29^ which commonly target a single molecule among MMPs and ADAMTS. However, at present, TNF-*α*-responsive miRNAs probably associated with OA development are poorly understood.

In this study, we explore TNF-*α*-responsive miRNAs in chondrocytes and attempt to identify miRNAs intimately implicated in TNF-*α*-mediated signaling and investigate their potential roles in cartilage matrix degradation during OA pathogenesis. Our results show that miR-145 is sensitive to TNF-*α*, characterized by a rapid reduction upon TNF-*α* stimulation. In turn, miR-145 suppresses TNF-*α*-mediated JNK/c-Jun and p38/ATF2 activation and induction of MMP-3, MMP-13, and Adamts-5 by directly targeting mitogen-activated protein kinase kinase 4 (MKK4), and consequently attenuates cartilage matrix degradation in experimental OA.

## Results

### MiR-145 is downregulated in TNF-*α*-stimulated chondrocytes and OA cartilage

**Figure 1:**
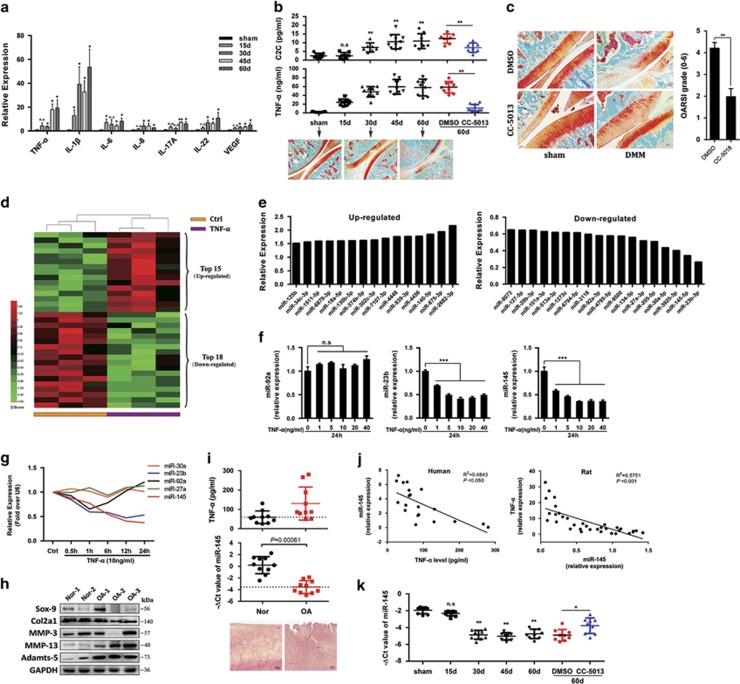
Expression level of miR-145 in TNF-*α*-stimulated chondrocytes and OA cartilage. (**a**) The mRNA levels of proinflammatory cytokines in rat cartilage obtained from the sham group (*n*=6) and DMM groups (*n*=6) 15, 30, 45, and 60 days after the surgery. (**b**) Production of TNF-*α* and C2C in rat serum obtained from the sham group (*n*=9), DMM groups (*n*=6–9), DMSO group (*n*=7–9), and CC-5013 group (*n*=7-10) were measured by ELISA. (**c**) Cartilage destruction and OARSI grade in sham- and DMM-operated (60 days) rat IA injected with CC-5013 or vehicle (*n*=8). (**d**) Heatmaps of miRNAs differentially expressed in TNF-*α*-stimulated chondrocytes. (**e**) Expression levels of the indicated miRNAs in TNF-*α*-stimulated chondrocytes. (**f**) Quantitative real-time PCR (qRT-PCR) validation of miR-92a, miR-23b, and miR-145 expression in chondrocytes stimulated with TNF-*α* at different doses for 24 h. (**g**) Expression of the five miRNAs in chondrocytes stimulated with TNF-*α* for different time periods were analyzed by qRT-PCR. (**h** and **i**) Immunoblotting of catabolic and anabolic factors, expression of miR-145, ELISA results of TNF-*α*, and hematoxylin and eosin (HE) staining in cartilage from OA patients (*n*=3) or normal (*n*=2). Scale bar: 20 *μ*m. (**j**) Correlation between miR-145 and TNF-*α* in the cartilage of OA patients and DMM-operated rat. (**k**) Expression level of miR-145 in rat cartilage obtained from the sham group (*n*=6), DMM groups (*n*=6), DMSO group (*n*=6), and CC-5013 group (*n*=6). Data represent the mean±S.E.M. of at least *n*=4 independent experiments. *P<0.05, **P<0.01, ***P<0.001, NS=not significant

We first detected the levels of proinflammatory cytokines during surgery-induced OA and found that IL-1*β* and TNF-*α* were strongly enriched compared with other cytokines (Figure 1a). Treatment of chondrocytes with TNF-*α* promptly triggered the activation of NF-*κ*B and MAPK pathways (Supplementary Figure 1a), along with the nuclear import of p-p65, p-c-Jun, and p-ATF2 (Supplementary Figure 1b), resulting in the upregulation of MMP-3, MMP-13, and Adamts-5, which play crucial roles in OA cartilage destruction (Supplementary Figures 2a–g). Expectedly, blocking NF-*κ*B or MAPK partially restrained TNF-*α*-induced upregulation of matrix-degrading enzymes (Supplementary Figures 1c and d). Similarly, CC-5013 (TNF-*α* secretion inhibitor) reduced C2C (a cleavage neoepitope of collagen II) concentration, cartilage destruction, and the upregulation of MMP-3, MMP-13, and Adamts-5 during experimental OA (Figures 1b and c and Supplementary Figure 3a). Upon TNF-*α* stimulation, 15 miRNAs were upregulated more than 1.5-fold, whereas 18 miRNAs showed a >35% decrease (Figures 1d and e). Results of qRT-PCR validation showed that the expression of miR-23b and miR-145 were indeed reduced by TNF-*α* in time- and dose-dependent manners (Figures 1f and g). Of particular interest, miR-145 was selected for further investigation because of its higher expression level in chondrocytes and cartilage compared with miR-23b (Supplementary Figures 4a and b). In addition, miR-145 was markedly decreased in human OA cartilage, characterized by higher levels of TNF-*α*, MMP-3, MMP-13, and Adamts-5 and obvious destruction of cartilage matrix, compared with the normal cartilage (Figures 1h and i). Moreover, a solid inverse correlation was observed between miR-145 and TNF-*α* in surgery-induced OA and human OA cartilage (Figure 1j). Interestingly, CC-5013 significantly restored the reduction of miR-145 caused by destablization of the medial meniscus (DMM) surgery (Figure 1k). Altogether, these results demonstrate that miR-145 is negatively regulated by TNF-*α* in both chondrocytes and OA cartilage.

### The downregulation of miR-145 by TNF-*α* is dependent of the tradd–traf2–p65 axis

**Figure 2:**
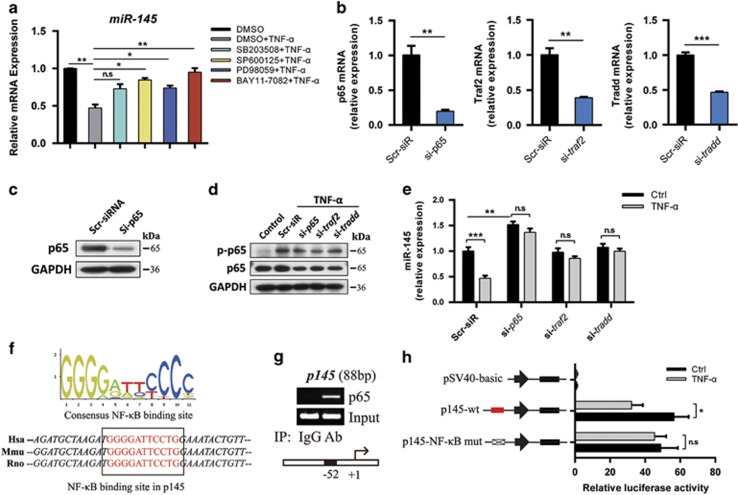
Requirement for NF-*κ*B-binding site in the regulation of miR-145. (**a**) Expression level of miR-145 in chondrocytes pretreated with MAPK inhibitors (SB203508, SP600125, or PD98059, 10 *μ*M) or NF-*κ*B inhibitor (BAY11-7082, 5 *μ*M) for 2 h and then cultured with or without TNF-*α* for 12 h. (**b**) The mRNA levels of p65, traf2, and tradd in chondrocytes transfected with *p65* siRNA, *traf2* siRNA, *tradd* siRNA, or negative control (scramble siRNA, Scr-siR). (**c**) The protein level of p65 in chondrocytes transfected with *p65* siRNA or Scr-siR. (**d**) Immunoblotting of p65 and p-p65 in chondrocytes transfected with siRNAs for *p65*, *traf2*, or *tradd*. (**e**) Expression level of miR-145 in chondrocytes transfected as described above and then cultured with or without TNF-*α*. (**f**) Human, mouse, and rat sequences of putative NF-*κ*B-binding sites (red) and their flanking regions in *miR-145* promoter. (**g**) Binding of NF-*κ*B subunit p65 to *miR-145* promoter was determined by the ChIP assay; normal rabbit IgG was used as the negative control. (**h**) Luciferase activity in lysates of SW1353 cells transfected with luciferase reporter plasmids of empty vector, *miR-145* promoter, or *miR-145* promoter with mutation of the p65-binding site, and then left unstimulated or stimulated with TNF-*α* for 12 h. Results were presented relative to *Renilla* luciferase activity. Data represent the mean±S.E.M. of at least *n*=4 independent experiments. *P<0.05, **P<0.01, ***P<0.001, NS=not significant

To elucidate the underlying mechanism of miR-145 reduction by TNF-*α*, NF-*κ*B and MAPK inhibitors were used. Results showed that BAY11-7082 (NF-*κ*B inhibitor) extremely rescued the inhibitory effects of TNF-*α* on miR-145 expression (Figure 2a). We then performed RNA interference (RNAi)-mediated knockdown of p65, traf2, and tradd in chondrocytes (Figures 2b–d). Indeed, knockdown of p65, traf2, or tradd blocked TNF-*α*-induced downregulation of miR-145 (Figure 2e). Bioinformatic analyses reveal that there is a consensus NF-*κ*B-binding site in *miR-145* promoter (Figure 2f). Thus, we performed chromatin immunoprecipitation (ChIP) assay and confirmed that TNF-*α* stimulation led to recruitment of NF-*κ*B subunit, p65, to the promoter of *miR-145* (Figure 2g). In cells expressing a luciferase construct containing the wild-type *miR-145* promoter, TNF-*α* markedly reduced luciferase activity, whereas mutation of the NF-*κ*B-binding site in the promoter inhibited TNF-*α*-triggered suppression of reporter activity (Figure 2h), indicating that p65 binding to the *miR-145* promoter is crucial for the reduction of miR-145 by TNF-*α*. These results collectively suggest that reduction of miR-145 by TNF-*α* is mainly through the canonical NF-*κ*B signaling pathway.

### MiR-145 inhibits TNF-*α*-induced expression of matrix-degrading enzymes

**Figure 3:**
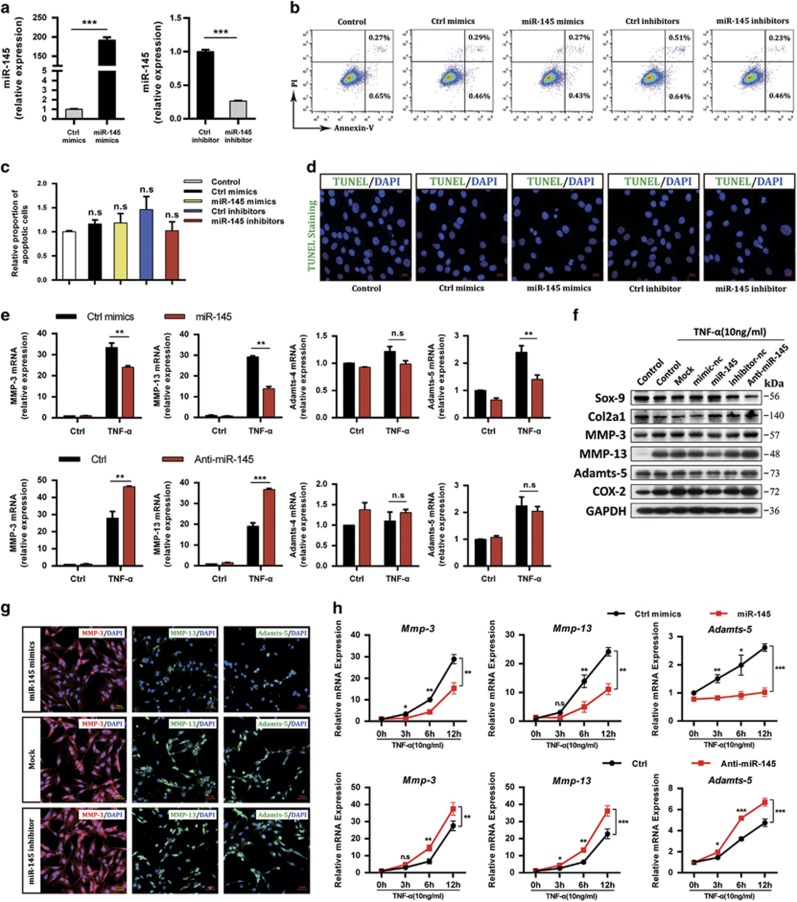
MiR-145 represses TNF-*α*-induced expression of matrix-degrading enzymes in chondrocytes. (**a–d**) Chondrocytes were transfected with miR-145 mimics, inhibitor, or their negative controls at a final concentration of 40 nM. At 24 h after transfection, (**a**) the expression level of miR-145 was measured by qRT-PCR and normalized to U6. Cell apoptosis was detected by (**b**) Annexin V-FITC/propidium iodide double staining with FACS analysis and (**d**) TUNEL staining. Scale bar: 10 *μ*m. (**c**) Quantitative results of the FACS analysis. (**e**) The mRNA and (**f**) protein levels of MMP-3, MMP-13, and Adamts-5 in chondrocytes transfected with miR-145 mimics, inhibitor, or their negative controls, and then cultured with or without TNF-*α*. (**g**) Immunofluorescence of MMP-3, MMP-13, and Adamts-5. Scale bar: 50 *μ*m. (**h**) The mRNA levels of MMP-3, MMP-13, and Adamts-5 in chondrocytes transfected as described above and then stimulated with TNF-*α* for different time periods as indicated. Data represent the mean±S.E.M. of at least *n*=4 independent experiments. *P<0.05, **P<0.01, ***P<0.001, NS=not significant

To assess the involvement of miR-145 in the regulation of TNF-*α*-induced expression of matrix-degrading enzymes, we transfected chondrocytes with miR-145 mimics or inhibitor (Figure 3a and Supplementary Figures 5a and b). Neither overexpression nor inhibition of miR-145 had any effect on chondrocyte viability (Figures 3b–d). Notably, overexpression of miR-145 inhibited, whereas inhibition of miR-145 promoted TNF-*α*-induced MMP-3, MMP-13, and Adamts-5, but not Adamts-4 (Figures 3e–g). Unexpectedly, miR-145 exerted no effect on TNF-*α*-induced downregulation of Sox-9, Col2a1, and Aggrecan in our experiments (Supplementary Figure 6a). Chondrocytes stimulated with TNF-*α* for different time periods were used to further characterize the inhibitory effect of miR-145 on TNF-*α*-induced matrix-degrading enzymes, and the results agreed with the findings above (Figure 3h). Additionally, we examined the effects of other four downregulated miRNAs (miR-23b, miR-92a, miR-27a, and miR-30a; results from microarray). However, all of them exerted no influence on TNF-*α*-induced upregulation of MMP-3, MMP-13, and Adamts-5 (Supplementary Figures 7a–e). These data clearly show that miR-145 inhibits TNF-*α*-induced upregulation of MMP-3, MMP-13, and Adamts-5 in chondrocytes.

### MiR-145 directly targets MKK4

**Figure 4:**
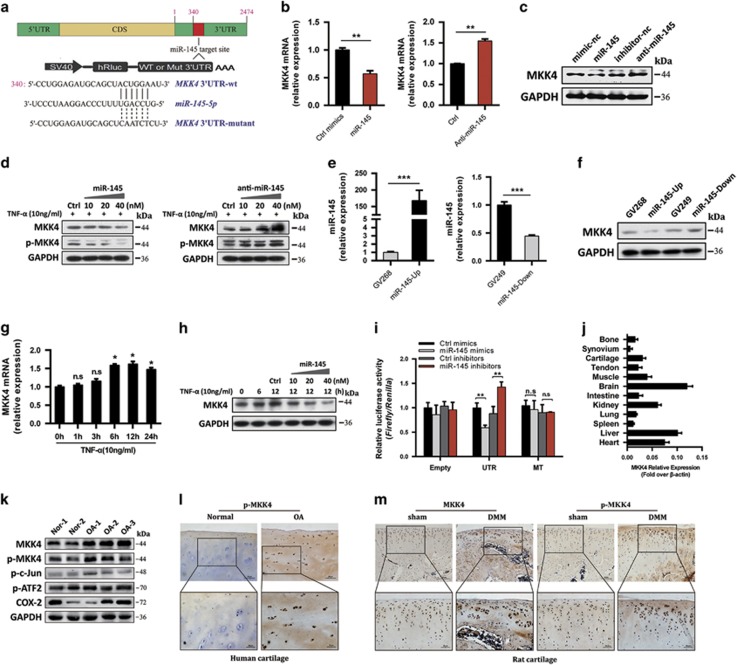
MiR-145 directly targets MKK4. (**a**) A schematic illustrating the design of luciferase reporters with the WT *MKK4* 3′-UTR or the site-directed mutant *MKK4* 3′-UTR. (**b** and **c**) Expression level of MKK4 in chondrocytes transfected with miR-145 mimics, inhibitor or their negative controls. (**d**) Immunoblotting of MKK4 and p-MKK4 in chondrocytes transfected at different doses (10, 20, and 40 nM) and then stimulated with TNF-*α*. (**e** and **f**) Expression level of miR-145 and MKK4 in chondrocytes transfected with miR-145 overexpression plasmid, inhibition plasmid or empty plasmids (GV268, GV249). (**g**) The mRNA level of MKK4 in chondrocytes stimulated with TNF-*α* for different time points. (**h**) Immunoblotting of MKK4 in chondrocytes transfected with miR-145 mimics at different doses (10, 20, and 40 nM) and then stimulated with TNF-*α*. (**i**) Effect of miR-145 mimics or inhibitor on the luciferase activity of WT *MKK4* 3′-UTR (UTR) or MUT *MKK4* 3′-UTR (MT) reporter in SW1353 cells. (**j**) MKK4 expression level in different tissues from rat. (**k**) Immunoblotting of MKK4 and its downstream molecules in cartilage samples from OA patients (*n*=3) or normal controls (*n*=2). (**l** and **m**) Immunostaining of MKK4 and p-MKK4 in human (*n*=6) and DMM-operated rat OA cartilage (*n*=6). Scale bar: × 100, 50 *μ*m; × 200, 20 *μ*m. Data represent the mean±S.E.M. of at least *n*=4 independent experiments. *P<0.05, **P<0.01, ***P<0.001, NS=not significant

Next, we investigated the possible targets of miR-145. Bioinformatic analysis using miRNA target prediction software revealed that MKK4, a member of the MKKs family, was a putative target of miR-145 (Figure 4a). Notably, miR-145 mimics reduced, whereas miR-145 inhibitor elevated, MKK4 and p-MKK4 in a dose-dependent manner under TNF-*α* stimulation (Figures 4b–d). Similarly, miR-145 overexpression plasmid inhibited, whereas miR-145 inhibition plasmid promoted, the expression of MKK4 (Figures 4e and f). We further observed that the endogenous MKK4 significantly increased within a short period upon TNF-*α* stimulation (Figure 4g), consistent with the rapid reduction of internal miR-145 level by TNF-*α*, and this effect was remarkably diminished by external miR-145 mimics at different doses (Figure 4h). To obtain more direct evidence, luciferase reporter constructs were generated and co-transfected with miR-145 mimics or inhibitors into the SW1353 cell line. Results showed that miR-145 mimics significantly inhibited, whereas miR-145 inhibitors enhanced, the luciferase activity of cells transfected with wild-type *MKK4* 3′-UTR, but not cells transfected with the mutant *MKK4* 3′-UTR (Figure 4i). We then assessed MKK4 expression profile in different tissues from rats. Despite the expression level of MKK4 in the brain, liver, and heart were much higher, MKK4 was found to be substantially expressed in articular cartilage compared with other joint tissues (bone, synovium, and tendon), which suggest that MKK4 may have a functional role in cartilage under physiological or pathological conditions (Figure 4j). Furthermore, MKK4 and its downstream molecules was markedly elevated in OA-affected, damaged regions of human cartilage (Figures 4k and l) and surgery-induced OA cartilage (Figure 4m), supporting a potential role of MKK4 in OA pathogenesis. Collectively, these results suggest that MKK4 is a direct target of miR-145 and that the endogenous MKK4 is tightly regulated by miR-145 in chondrocytes during TNF-*α* stimulation and OA pathogenesis.

### MiR-145 negatively regulates TNF-*α*-mediated signaling

**Figure 5:**
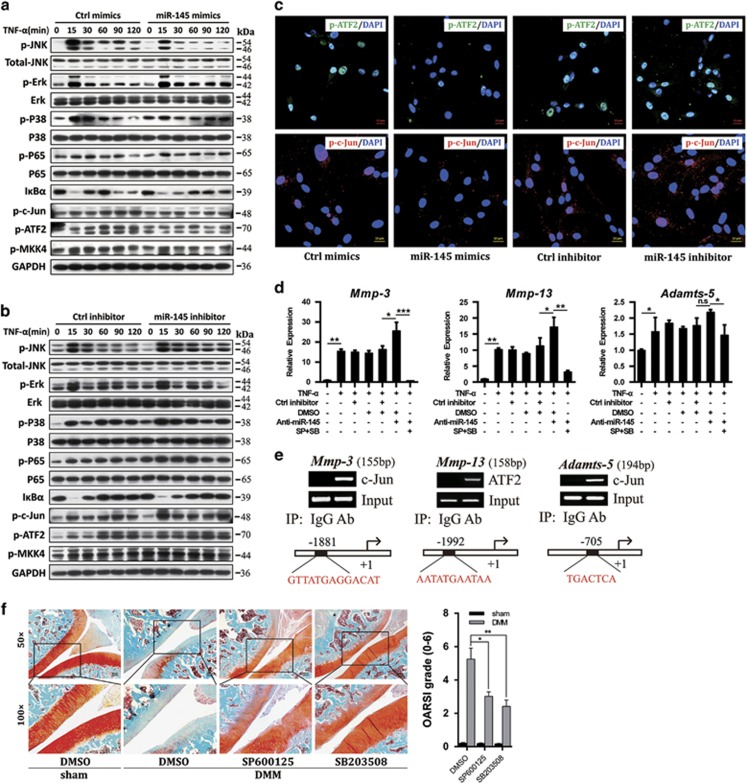
MiR-145 suppresses TNF-*α*-induced activation of JNK and p38 pathways. (**a** and **b**) Chondrocytes were transfected with miR-145 mimics, inhibitor, or their negative controls and then stimulated with TNF-*α* for different time periods as described. Protein levels of the main molecules involved in the NF-*κ*B and MAPK signaling pathways were analyzed by immunoblotting. (**c**) The nuclear import of p-c-Jun and p-ATF2 induced by TNF-*α* in chondrocytes transfected with miR-145 mimics, inhibitor, or their negative controls. Scale bar: 10 *μ*m. (**d**) The mRNA levels of MMP-3, MMP-13, and Adamts-5 in chondrocytes transfected with miR-145 inhibitor alone or in combination with SB203508 and SP600125 and then treated with TNF-*α*. (**e**) ChIP assays for binding of c-Jun or ATF2 to the promoter region of *MMP-3*, *MMP-13*, or *Adamts-5* in TNF-*α*-treated chondrocytes. Normal rabbit IgG was used as the negative control. (**f**) Safranin-O staining and OARSI grade in sham- and DMM-operated rat IA injected with SP600125, SB203508 or vehicle (*n*=8). Scale bar: × 50, 100 *μ*m; × 100, 50 *μ*m. Data represent the mean±S.E.M. of at least *n*=4 independent experiments. *P<0.05, **P<0.01, ***P<0.001, NS=not significant

To elucidate the underlying mechanism through which miR-145 inhibits the production of TNF-*α*-induced matrix-degrading enzymes, we assessed the effect of miR-145 on the activation of NF-*κ*B and MAPK pathways. Data showed that the levels of p-Erk, p-p65, and I*κ*B*α* were comparable; however, miR-145 mimics greatly repressed, by contrast, miR-145 inhibitor enhanced, the phosphorylation of MKK4, JNK, p38, c-Jun, and ATF2 under TNF-*α* stimulation (Figures 5a and b). Furthermore, miR-145 mimics additionally inhibited, whereas miR-145 inhibitor promoted, the nuclear import of p-c-Jun and p-ATF2 induced by TNF-*α* (Figure 5c). It is worth noting that the facilitation of miR-145 inhibitor on TNF-*α*-induced upregulation of MMP-3, MMP-13, and Adamts-5 was partially blocked by JNK- and p38-specific inhibitors (Figure 5d), which indeed effectively inhibited the phosphorylation of JNK or p38, as well as their downstream cascades, alone or in combination (Supplementary Figures 8a–c). ChIP assay in TNF-*α*-stimulated chondrocytes revealed that ATF2 interacted with a binding motif (5′-AATATGAATAA-3′) in the promoter region of *Mmp-13*, whereas c-Jun bound to promoters of *Mmp-3* and *Adamts-5* (Figure 5e), suggesting direct modulation of these genes by c-Jun or ATF2. Moreover, JNK- or p38-specific inhibitors effectively attenuated cartilage destruction in DMM-operated rat (Figure 5f). In summary, these observations strongly suggest that miR-145 has a general inhibitory role in TNF-*α*-mediated JNK/c-Jun and p38/ATF2 activation and downstream gene induction.

### MKK4 critically controls catabolic gene expression and OA pathogenesis

**Figure 6:**
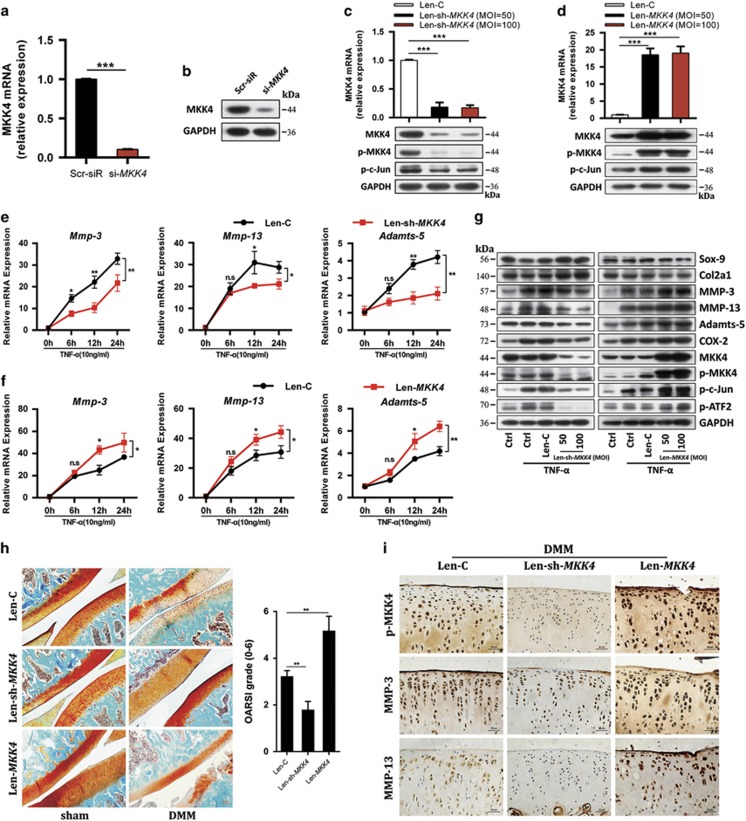
MKK4 regulates TNF-*α*-triggered matrix-degrading enzymes and cartilage degradation. (**a** and **b**) The mRNA and protein levels of MKK4 in chondrocytes transfected with Scr-siR or *MKK4* siRNA (#1). (**c** and **d**) The mRNA level of MKK4 and protein levels of MKK4, p-MKK4, and p-c-Jun in chondrocytes infected with Len-C (lentivirus containing empty vector), Len-sh-*MKK4* (lentivirus containing sh-*MKK4*), or Len-*MKK4* (lentivirus containing the entire CDS sequence of *MKK4*) at the indicated multiplicity of infection (MOI=50 or 100). (**e**–**g**) Chondrocytes were infected with Len-C, Len-sh-*MKK4*, or Len-*MKK4* (100 MOI). At 48 h after infection, the cells were stimulated with TNF-*α*. The mRNA levels of MMP-3, MMP-13, and Adamts-5 were evaluated by qRT-PCR. The protein levels of catabolic factors, anabolic factors, MKK4, and its downstream molecules were measured by immunoblotting. (**h**) Safranin-O staining and OARSI grade in sham- and DMM-operated rat IA injected with Len-C, Len-sh-*MKK4*, or Len-*MKK4* (*n*=8). Scale bar: 50 *μ*m. (**i**) Immunostaining of p-MKK4, MMP-3, and MMP-13 in cartilage tissue of sham-operated and DMM-operated rat IA injected with Len-C, Len-sh-*MKK4*, or Len-*MKK4* (*n*=8). Scale bar: 20 *μ*m. Data represent the mean±S.E.M. of at least *n*=4 independent experiments. *P<0.05, **P<0.01, ***P<0.001, NS=not significant

To further confirm the role of MKK4 in TNF-*α*-mediated signaling and induction of downstream genes, we generated siRNAs for MKK4 and HGK, a MAP kinase kinase kinase that specifically activates MKK4 (Supplementary Figures 9a and b). siRNA#1 for MKK4 and siRNA#3 for HGK were used for the following investigations (Figures 6a and b), both of which efficiently inhibited the phosphorylation of JNK and p38, as well as their downstream cascades (Supplementary Figure 9c). Interestingly, knockdown of MKK4 or HGK suppressed TNF-*α*-induced upregulation of MMP-3, MMP-13, Adamts-5, and COX-2 (Supplementary Figures 9d and e). For a more durable effect *in vitro* and *in vivo*, lentivirus containing MKK4 short hairpin RNA plasmid (Len-sh-*MKK4*), or the entire CDS sequence of MKK4 (Len-*MKK4*) was generated. Indeed, Len-sh-*MKK4* infection effectively depleted, whereas Len-*MKK4* elevated MKK4, p-MKK4, and p-c-Jun (Figures 6c and d). As expected, Len-sh-*MKK4* significantly abrogated, whereas Len-*MKK4* enhanced, TNF-*α*-mediated JNK/c-Jun and p38/ATF2 activation, with concomitant alteration of MMP-3, MMP-13, and Adamts-5 (Figures 6e–g). In addition, Len-sh-*MKK4* also abrogated the synergistically enhanced TNF-*α*-induced upregulation of MMP-3, MMP-13, and Adamts-5 caused by miR-145 inhibitor (Supplementary Figure 10a); meanwhile, Len-*MKK4* completely abolished the inhibitory effect of miR-145 mimics on TNF-*α*-induced upregulation of catabolic genes (Supplementary Figure 10b), which further confirmed that the inhibitory effect of miR-145 on TNF-*α* is mediated by MKK4. The role of MKK4 in OA pathogenesis was examined via IA injection of Len-sh-*MKK4* or Len-*MKK4* into rat knee joints. Efficient lentiviral infection in rat articular cartilage was confirmed by the expression of green fluorescence protein (GFP) via IA injection of lentivirus carrying the GFP gene (Len-GFP), and found that Len-sh-*MKK4* injection significantly reduced MKK4 in all joint tissues, except for meniscus (Supplementary Figures 11a–c), compared with Len-C, IA injection of Len-sh-*MKK4* effectively attenuated cartilage destruction caused by DMM surgery, along with downregulation of p-MKK4, MMP-3, and MMP-13 in cartilage; by contrast, Len-*MKK4* obviously exacerbated cartilage destruction, with concomitant upregulation of p-MKK4, MMP-3, and MMP-13 (Figures 6h and i). These data solidly confirm that MKK4 has an essential role in miR-145-mediated suppression of TNF-*α*-induced catabolic factor expression.

### MiR-145 attenuates cartilage matrix degradation in experimental OA likely through its suppression of MKK4-mediated TNF-*α* signaling

**Figure 7:**
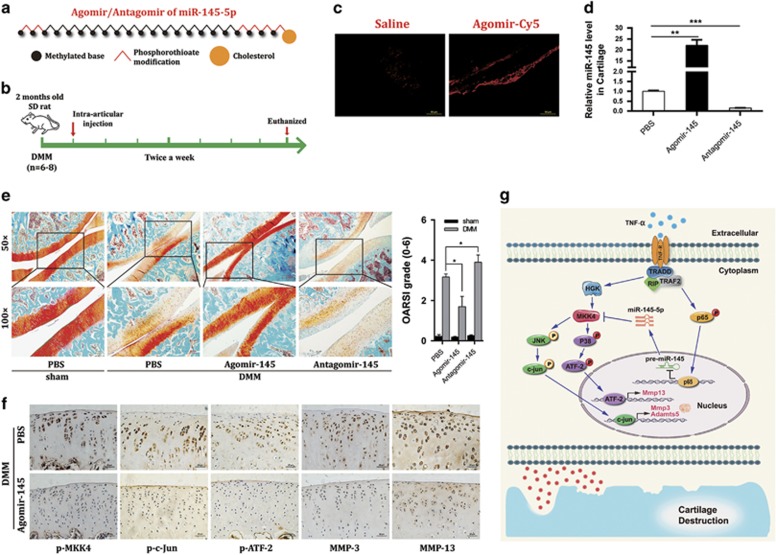
MiR-145 counteracts cartilage matrix degradation in surgery-induced OA. (**a**) The diagrammatic sketch of agomir/antagomir-145 with specific chemical modifications. (**b**) Schematic of the animal experiments (for each group, *n*=6, rat). (**c** and **d**) Transfection efficiency of agomir labeled by cy5 in articular cartilage or chondrocytes compared with the saline group (*n*=6). Scale bar: 50 *μ*m. (**e**) Safranin-O staining and OARSI grade in sham-operated and DMM-operated rat IA injected with agomir-145, antagomir-145, or PBS (*n*=8). Scale bar: × 50, 100 *μ*m; × 100, 50 *μ*m. (**f**) Immunostaining of MMP-3, MMP-13, p-MKK4, p-c-Jun, and p-ATF2 in cartilage tissue of sham- and DMM-operated rat IA injected with agomir-145, antagomir-145, or PBS (*n*=8). Scale bar: 20 *μ*m. (**g**) Diagram depicting the signaling pathways for miR-145 in the regulation of TNF-*α*-induced expression of matrix-degrading enzymes in chondrocytes. P, phosphorylation. Data represent the mean±S.E.M. of at least *n*=4 independent experiments. *P<0.05, **P<0.01, ***P<0.001, NS=not significant

Considering that miR-145 has an inhibitory role in TNF-*α*-mediated signaling and induction of matrix-degrading enzymes *in vitro*, we further examined whether miR-145 affects catabolic genes expression and subsequent OA pathogenesis *in vivo*. For a more durable effect of miR-145 *in vivo*, we generated agomir-145 and antagomir-145 via several specific chemical modifications (Figure 7a). Subsequently, agomir-145, antagomir-145, or an equivalent volume of vehicle was IA injected into the knee joint of rats with surgery-induced OA (Figure 7b). Both agomir-145 and antagomir-145 could easily enter chondrocytes *in vitro* (Supplementary Figures 11d and e) and *in vivo* (Figures 7c and d) without any carrier. Remarkably, IA injection of agomir-145 effectively alleviated, whereas antagomir-145 worsened, cartilage destruction triggered by DMM surgery (Figure 7e). Meanwhile, injection of agomir-145 suppressed the expression of MMP-3 and MMP-13, as well as p-MKK4, p-c-Jun, and p-ATF2 in cartilage tissue after surgery (Figure 7f), indicating that MKK4-JNK/p38-c-Jun/ATF2-mediated suppression of matrix-degrading enzymes expression is mainly responsible for the observed inhibitory effect of miR-145 on cartilage destruction caused by OA (Figure 7g). Thus, our results support the utility of miR-145 as an effective therapeutic target for cartilage matrix degradation in OA.

## Discussion

IL-1*β* and TNF-*α* are the major proinflammatory cytokines in OA. Several miRNAs involved in IL-1*β*-mediated signaling during OA have been reported. MiR-146a, an IL-1*β*-responsive miRNA, impairs TGF-*β* signaling pathway through the targeted inhibition of Smad4 in cartilage.^26^ MiR-27b and miR-127 inhibit the IL-1*β*-induced upregulation of MMP-13 in human osteoarthritic chondrocytes.^24, 30^ However, no report on TNF-*α*-responsive miRNA in chondrocytes was found. In this study, we initially identified miR-145 as a TNF-*α*-responsive miRNA in chondrocytes, characterized by a rapid reduction upon TNF-*α* stimulation *in vitro* and a negative correlation with the secretion of TNF-*α* during experimental OA *in vivo*. Moreover, blocking the canonical NF-*κ*B pathway or RNAi-mediated knockdown of tradd, traf2, or p65 partially rescued the reduction of miR-145 by TNF-*α*, suggesting that the inhibitory effect of TNF-*α* on miR-145 is mediated by p65.

Previous studies have reported several miRNAs involved in OA pathogenesis. MiR-140, a cartilage-specific miRNA, regulates the expression of Adamts-5 in chondrocytes,^23^ and *miR-140*-/- mice display an OA-like phenotype.^31^ MiR-27a affects the expression of MMP-13 and IGFBP-5.^32^ MiR-93 regulates collagen loss by targeting MMP-3,^33^ and miR-125b regulates the expression of Adamts-4 in human chondrocytes.^34^ Apparently, the OA-related miRNAs mentioned above merely target a single molecule among MMPs and ADAMTS. However, in the present study, we found that miR-145 overexpression could simultaneously suppress several matrix-degrading enzymes at transcriptional and post-transcriptional levels. In addition, neither overexpression nor inhibition of miR-145 affected the viability of chondrocytes. Thus, miR-145 may be a more effective target for OA treatment because of its broad inhibition of matrix-degrading enzymes production caused by TNF-*α*. Unexpectedly, miR-145 exerted no effect on anabolic factors in chondrocytes treated with TNF-*α*. Consistently, our further studies confirm that the promoter regions of *Mmp-3*, *Mmp-13* and *Adamts-5* but not *Sox-9*, *Col2a1*, and *Aggrecan*, harbor the c-Jun or ATF2-binding motif; therefore, MMP-3, MMP-13, and Adamts-5 but not Sox-9, Col2a1, and Aggrecan can be directly modulated by the miR-145/MKK4-JNK/p38-c-Jun/ATF2 axis.

Our results demonstrated that miR-145 was sensitive to TNF-*α*, characterized by a rapid reduction upon TNF-*α* stimulation and a solid negative correlation with TNF-*α* secretion during the progression of human and experimental OA, implying that miR-145 is possibly involved in OA pathogenesis. Indeed, modulation of miR-145 efficiently affected ECM degradation during OA, as evidenced by that miR-145 gain-of-function was able to restrain TNF-*α*-induced matrix-degrading enzymes and cartilage destruction caused by surgery-induced OA. Moreover, we uncovered the molecular mechanism underlying the protective effect of miR-145 on OA cartilage *in vitro* and further confirmed it *in vivo*. Collectively, miR-145 critically controls OA development through strictly modulating the transcription of several key TNF-*α*-induced matrix-degrading enzymes. On the other hand, miR-145 had also been identified to be involved in the regulation of chondrogenic differentiation in a previous study.^35^ Thus, we propose miR-145 as an important regulator of cartilage homeostasis, especially the catabolic signals, and its abnormal downregulation by TNF-*α* facilitates the progression of OA.

As is well known, the JNK and p38 MAPKs are activated by MKK4 and MKK7, and consequently capacitate the nuclear import of p-c-Jun and p-ATF2, eventually resulting in alteration of gene transcription.^36^ However, no report was found on the involvement of MKK4 or MKK7 in the modulation of MMPs and ADAMTS during OA. In the present study, we report for the first time that MKK4 is a conserved target gene of miR-145. Binding of miR-145 to *MKK4* 3′-UTR severly impaired the translation of MKK4, resulting in suppression of TNF-*α*-mediated signaling and induction of downstream matrix-degrading enzymes. Moreover, our results showed that MKK4 was significantly elevated in OA cartilage. Depletion of MKK4 or its upstream molecule HGK remarkably attenuated TNF-*α*-induced JNK/c-Jun and p38/ATF2 activation, and subsequently the production of matrix-degrading enzymes. In contrast, MKK4 overexpression accelerated TNF-*α*-mediated signaling activation and induction of catabolic genes. *In vivo*, we observed that blocking JNK or p38 signaling, or depletion of MKK4 partially, attenuated cartilage destruction caused by surgery-induced OA.

TNF-*α* is an important mediator of matrix degradation during OA, exerting its functions through inducing and sustaining the inflammatory cascades.^4^ Thus, negative regulation of TNF-*α* signaling is critical to shut off persistent inflammatory responses for the prevention of cartilage matrix degradation. So far, not much is known about how TNF-*α*-mediated signaling is negatively modulated. This study showed that miR-145 can be used as a potential novel target for therapy of OA because of its broadly inhibitory effects on TNF-*α*-mediated signaling and expression of MMPs and ADAMTS *in vitro* and *in vivo*, and more importantly, compared with the recombinant proteins, miR-145 agonist via methylated modifications exerts more stable and durable effect. It is also worth mentioning that miR-145 may also serve as an efficient therapeutic target in inflammatory arthritis such as rheumatoid arthritis (RA) in consideration of the similar inflammatory conditions in patients with RA.

In conclusion, we elucidate a novel regulatory mechanism by discovering that miR-145 is a crucial negative regulator of TNF-*α*-mediated signaling activation and induction of cartilage matrix degradation mechanically through the MKK4-JNK/p38-c-Jun/ATF2 axis during OA pathogenesis, and demonstrate the potential utility of miR-145 and MKK4 as therapy targets for OA. To our knowledge, miR-145 represents the first identified TNF-*α*-reduced miRNA, which in turn serves as a negative regulator of TNF-*α*-mediated signaling activation and transcription.

## Materials and methods

### Human OA cartilage and experimental OA in rats

Human OA cartilage was sampled from OA patients (*n*=10) who underwent total joint replacement. Undamaged areas in the same patient were sampled as the normal cartilage. Ethical approval was obtained from the Medical Ethics Committee of Xin Hua Hospital, Shanghai Jiao Tong University School of Medicine, and informed consent was obtained from all participants. All experiments involving rats were purchased from Shanghai SLAC Laboratory Animal Co. Ltd (Shanghai, China), and animal handling and experimental procedures were performed following the approval from the Institute of Health Sciences Institutional Animal Care and Use Committee. Experimental OA in 12-week-old SD rat (male, 200 g) was induced by medial collateral ligament transection and DMM as described previously.^37, 38^ SP600125 (JNK inhibitor, 10 *μ*M) (S1460; Selleck, Houston, TX, USA), SB203508 (P38 inhibitor, 10 *μ*M) (S1076; Selleck), CC-5013 (TNF-*α* secretion inhibitor, 10 *μ*M) (S1029; Selleck), miR-145 agomir (50 *μ*M), miR-145 antagomir (200 *μ*M), and empty lentivirus (Len-C) or lentivirus (1 × 10^9^ PFU, 20 *μ*l) expressing sh-*MKK4* (Len-sh-*MKK4*) or MKK4 (Len-*MKK4*) or the equivalent volume of vehicle (DMSO or PBS) were IA injected into the knee joints of recipient rats 1 week after the surgery (20 *μ*l per joint per rat two times a week for 7 weeks) (*n*=6–10 per group). Rats were killed 8 weeks after the surgery, and samples of the knee joints were collected.

### Histology and immunostaining

Human OA cartilage was fixed in 4% paraformaldehyde and embedded in paraffin. Paraffin blocks were sectioned at a thickness of 5 *μ*m. Sections were deparaffinized in xylene, hydrated with graded ethanol, and stained with HE. Cartilage destruction of rat knee joints was examined using safranin-O staining and scored by two observers blinded to group-identifying information using the OARSI grading system.^39^ For immunohistochemistry, antigen retrieval was performed by incubating at 37 °C with 0.05% trypsin (pH 7.8). After blocking with 1% bovine serum albumin (BSA), sections were incubated at 4 °C overnight with primary antibodies against MKK4 (1 : 100; ab131351; Abcam, Cambridge, UK), phospho-MKK4 (1 : 50; sc-101795; Santa Cruz, Santa Cruz, CA, USA), phospho-c-Jun (1 : 100; ab13671; Abcam), phospho-ATF2 (1 : 200; 9221; Cell Signaling Technology, Danvers, MA, USA), MMP-3 (1 : 50; sc-6839; Santa Cruz), and MMP-13 (1 : 50; sc-30073; Santa Cruz).

### Primary culture of articular chondrocytes and transfection

Rat articular chondrocytes were isolated from femoral heads, femoral condyles, and tibial plateaus of rats as described previously.^40^ Cultured chondrocytes were maintained as a monolayer in DMEM/F12 (Gibco, Thermo Fisher Scientific, Waltham, MA, USA) with 10% fetal bovine serum (Gibco), 100 U/ml penicillin G, 100 *μ*g/ml streptomycin, and 2.5 *μ*g/ml amphotericin B under a humidified atmosphere of 5% CO_2_ in air at 37 °C. Recombinant TNF-*α* (10 ng/ml; 300-01A; PeproTech, Rocky Hill, NJ, USA) was used to stimulate chondrocytes in complete medium. miRNA mimics or inhibitor (GenePharma, Shanghai, China) and siRNAs (GenePharma) were transfected with Lipofectamine 2000 (Invitrogen, Carlsbad, CA, USA) according to the manufacturer’s instructions at a concentration of 40 nM. Sequences of miRNA mimics and inhibitor are shown in Supplementary Table 1.

### Microarray

The miRNA expression profiles of the primary rat chondrocytes treated with or without TNF-*α* (10 ng/ml) for 24 h were determined by miRNA microarray analysis (LC Sciences, Houston, TX, USA). Data were analyzed by first subtracting the background and then normalizing the signals using a LOWESS filter (locally weighted regression). Normalized data were further analyzed by two-tailed Student’s *t*-test. miRNAs with *P*<0.05 were considered differentially expressed (shown in Supplementary Table 2). In consideration of the modest elevated levels of upregulated miRNAs, we focused on the downregulated miRNAs. After assessing the cross-species conservation, five downregulated miRNAs (with maximal fold change) were selected for qRT-PCR validation. Finally, miR-145 was selected for further investigation because of its higher expression level in chondrocytes and cartilage compared with other miRNAs.

### miRNA target prediction

The miRNA target interaction was predicted by miRanda. The TargetScan and miRDB were also applied to validate the interaction. Targets (MKK4, ROCK1, NEDD9, SOX9, and SMAD3) with high score (>100) were considered as candidate genes. The miRWalk database was further applied to exclude the genes that had been validated as targets of miR-145 in previous reports. The expression level of candidate genes in chondrocytes and cartilage were also evaluated by real-time PCR. Altogether, MKK4 was selected for further investigation.

### Construction of plasmids and 3′-UTR cloning

The miR-145 expression plasmid was created as follows: the precursor sequence for miR-145 was cloned into the XhoI/*Kpn*I site of the GV268 vector using the following primers: 5′-ACGGGCCCTCTAGACTCGAGCATATAGCACCCCACACTG-3′ (sense) and 5′-TTAAACTTAAGCTTGGTACCGAGGTCCCAAGACCGCTTAC-3′ (antisense). The miR-145 inhibition plasmid was generated by the reverse complementary sequence of mature miR-145: 5′-AGGGATTCCTGGGAAAACTGGAC-3′, which was cloned into the *Bam*HI/*Hind*III site of the GV249 vector. The following primers were used: 5′-AGCTAAAAAGTCCAGTTTTCCCAGGAATCCCTG-3′ (sense) and 5′-GATCCAGGGATTCCTGGGAAAACTGGACTTTTT-3′ (antisense). Fragment harboring the 3′-UTR of MKK4 containing the miR-145-binding sequence were cloned into the *Xba*I/*Xba*I site of the GV249 vector using the following primers: 5′-GATCGCCGTGTAATTCTAGAACATATTCATGAAATGTGG-3′ (sense) and 5′-CCGGCCGCCCCGACTCTAGAGCTGTGCAAGCTCTTCTC-3′ (antisense). For promoter reporter assay, fragment harboring the promoter of miR-145 containing the NF-*κ*B-binding site (5′-GGGGATTCCTG-3′) was cloned into the *Xho*I/*Hind*III site of the basic vector (Promega, Sunnyvale, CA, USA) using the following primers: 5′-CCGCTCGAGCGGTCACGGTCCAGTTTTCCCAGGAATCC-3′ (sense) and 5′-CCCAAGCTTGGGCCATGACCTCAAGAACAGTATTTC-3′ (antisense). Site-specific mutant were generated by site-directed mutagenesis using the QuikChange Site-Directed Mutagenesis Kit (Stratagene, La Jolla, CA, USA). All sequences of the amplified products were confirmed by DNA sequencing.

### Luciferase reporter assay

All plasmids for transfection were prepared using the Qiagen Plasmid Purification Kit (Qiagen, Hilden, Germany). The SW1353 cell line were co-transfected with miR-145 mimics or inhibitor (40 nM), luciferase constructs (described above) (200 ng), and pRL-TK (Promega) *Renilla* luciferase plasmid (50 ng). For promoter reporter assay, SW1353 cells were transfected with a mixture of 200 ng luciferase vector (containing p145-wt or p145-NF-*κ*B-mut) and 50 ng pRL-TK *Renilla* luciferase plasmid. Luciferase assays were performed with the dual-luciferase reporter assay system (E1910; Promega) according to the manufacturer’s instructions. Luminescent signals were quantified by a luminometer (Glomax; Promega), and each value from the *Renilla* luciferase construct was normalized by firefly luciferase.

### Immunofluorescence

Chondrocytes were grown on a 20 mm glass bottom cell culture dish (801001; Nest Biotechnology Co. Ltd, Shanghai, China). Cells were fixed with 4% paraformaldehyde for 15 min, washed two times with PBS containing 0.05% Tween-20, permeabilized with 0.3% Triton X-100 for 5 min, and then blocked with 1% BSA for 30 min. Then, the cells were incubated at 4 °C overnight with the appropriate primary antibodies. Subsequently, the cells were washed with PBS and incubated for 1 h at room temperature with Alexa Fluor 488- or 594-conjugated goat anti-rabbit or donkey anti-mouse IgG secondary antibody (Invitrogen). 4′,6-Diamidino-2-phenylindole (Life Technologies, Carlsbad, CA, USA) was used for nuclear staining. Cells were visualized under a confocal microscope (Leica TCSSP5; Leica, Wetzlar, Germany).

### RNA interference

RNAi was performed using siGENOME SMART pool siRNA (Dharmacon, Lafayette, CO, USA) targeting rat MKK4 (gene ID 287398), HGK (gene ID 301363), p65 (gene ID 25716), Traf2 (gene ID 311786), and Tradd (gene ID 246756). The siRNA sequences are described in Supplementary Table 3.

### Lentivirus infection

Chondrocytes were infected with Len-C, Len-sh-MKK4, and Len-MKK4 in combination with polybrene at the indicated MOI for 12 h, washed, and then maintained for 48–72 h before further analysis according to the manufacturer’s instructions. For Len-sh-MKK4 construction, LV3 (pGLVH1/GFP+Puro) (GenePharma Co., Ltd, Shanghai, China) was used. The following primers were used: 5′-GATCCGGCAGATAATGGCAGTTAATTCAAGAGATTAACTGCCATTATCTGCCTTTTTTG-3′ (sense) and 5′-AATTCAAAAAAGGCAGATAATGGCAGTTAATCTCTTGAATTAACTGCCATTATCTGCCG-3′ (antisense). For Len-MKK4, the PCR products of MKK4 were cloned into the *Not*I/*Bam*HI site of LV-5 (pEF-1a/GFP+Puro) using the following primers: 5′-TTAAGCTTATGGCGGCTCCGAGCC-3′ (sense) and 5′-CGGGATCCTCAGTCGACATACATGGGA-3′ (antisense).

### Apoptosis of chondrocytes

Chondrocyte apoptosis after transfection was determined by TUNEL assay using a kit (C1086; Beyotime Biotechnology, Jiangsu, China) according to the manufacturer’s instructions. Specimens were visualized under a confocal microscope (Leica). Chondrocytes after transfection were also detected by Annexin V-FITC/propidium iodide double staining (V13241; Life Technologies) with FACS analysis.

### Real-time PCR

Total RNA from tissues or cells was extracted using Trizol reagent (Invitrogen). First-strand cDNA was synthesized from 1 mg of total RNA by incubating for 1 h at 42 °C using the RevertAid Reverse Transcriptase (EP0442; Thermo Scientific, Waltham, MA, USA) following oligonucleotide (dT) priming in accordance with the manufacturer’s instructions. qRT-PCR was performed by ViiATM 7 Real-Time PCR System (Life Technologies) using SYBR Premix Ex Taq (Takara, Dalian, China). Data were analyzed using the comparison Ct (2^−ΔΔCt^) method. GAPDH and small nuclear RNA U6 were used as internal controls for cDNA and miRNA, respectively. The data in Figure 4j was normalized by *β*-actin, due to the low uniformity of GAPDH among the different tissues. Each experiment was performed in triplicate. The primer sequences used in this study are summarized in Supplementary Table 4.

### Immunoblotting

Cells were lysed on ice for 30 min in lysis buffer containing 50 mM Tris-HCl, pH 7.4, 150 mM NaCl, 1% Nonidet P-40, and 0.1% SDS supplemented with protease inhibitors (10 mg/ml leupeptin, 10 mg/ml pepstatin A, and 10 mg/ml aprotinin). Protein fractions were collected by centrifugation at 15 000 × *g* at 4 °C for 10 min, subjected to 10% SDS-PAGE, and then electrotransferred onto nitrocellulose membranes (Whatman, Piscataway, NJ, USA). The membranes were blocked with 5% BSA and then incubated with specific antibodies overnight at 4 °C. The primary antibodies were from the following sources: Sox-9 (1 : 500, ab26414; Abcam), Col2a1 (1 : 1000, BS1071; Bioworld Technology, St. Louis Park, MN, USA), MMP-3 (1 : 200; sc-6839; Santa Cruz), MMP-13 (1 : 200; sc-30073; Santa Cruz), Adamts-5 (1 : 200; sc-83186; Santa Cruz), COX-2 (1 : 500, ab15191; Abcam), JNK, phospho-JNK, Erk, phosphor-Erk, p38, phospho-p38, p65, phospho-p65, I*κ*B*α*, phospho-c-Jun, phosphor-ATF2, MKK4, phospho-MKK4, HGK (1 : 1000; all from Cell Signaling Technology), and GAPDH (1 : 5000, G9545  Sigma-Aldrich, St. Louis, MO, USA). HRP-conjugated secondary antibodies (Cell Signaling Technology) were used at a 1:1000 dilution. The antigen–antibody complexes were visualized using the enhanced chemiluminescence detection system (Millipore, Darmstadt, Germany) as recommended by the manufacturer.

### Chromatin immunoprecipitation

ChIP assay was performed using EZ ChIP Chromatin Immunoprecipitation Kit (Millipore) in accordance with the manufacturer’s instructions. Immunoprecipitation was carried out overnight with p65 (8242 S; Cell Signaling Technology), c-Jun (9165; Cell Signaling Technology), and ATF2 (8638; Cell Signaling Technology) antibodies or normal rabbit IgG as a negative control. Protein A/G agarose was used to pull down the antigen–antibody compounds and then washed four times with washing buffers. The DNA–protein crosslinks were reversed with 5 M NaCl at 65 °C for 6 h, and DNA from each sample was purified. PCR was performed with 2 *μl* of DNA samples with the primers shown in Supplementary Table 4. The PCR products were analyzed by 2% agarose gel electrophoresis.

### Enzyme-linked immunosorbent assay

The cytokine and the degradation products of collagen II (C2C) production from the samples of human OA cartilage and surgery-induced OA in rats were assessed with TNF-*α* (560479; BD Biosciences, San Jose, CA, USA) or C2C (YM8649; Yuan Mu Bioscience, Shanghai, China) ELISA Kits (enzyme-linked immunosorbent assay). A standard curve was generated using known concentrations of the respective purified recombinant TNF-*α* or C2C.

### Statistical analysis

All statistical analyses were performed with SPSS 19.0 software (SPSS Inc., IBM Corporation, Armonk, NY, USA). Data are presented as mean±S.D. Statistical differences between two groups were determined by two-tailed Student’s *t*-test. *P*<0.05 was considered statistically significant.

## Publisher’s Note

Springer Nature remains neutral with regard to jurisdictional claims in published maps and institutional affiliations.
