# Supplementary material for: MicroRNA-145 attenuates TNF-α-driven cartilage matrix degradation in osteoarthritis via direct suppression of MKK4
Source: Cell Death Dis. 2017 Oct 26;8(10):e3140–. doi: 10.1038/cddis.2017.522 (PMC5682684; doi:10.1038/cddis.2017.522)
Supplement: Supplementary Information [file cddis2017522x1.docx]

**Supplementary information**

**MicroRNA-145 attenuates TNF-α-driven** **cartilage matrix degradation in osteoarthritis via direct suppression of MKK4**

Guo-li Hu^1^, Xiao-ying Zhao^1^, Chuan-dong Wang^1^, Yi-yun Geng^1^, Jing-yu Zhao^1^, Jia-jia Xu^1^, Bin Zuo^2^, Chen Zhao^3^, Cheng-long Wang^2^, Xiao-ling Zhang^1, 2*^

Supplementary Tables 1-4

Supplementary Figures 1-11

**Supplementary Table 1.** Sequences of miRNA mimics and inhibitor

| **miRNAs** | **Sequence** | **Spezies** |
| --- | --- | --- |
| **mimics N.C** | 5’-UUGUACUACACAAAAGUACUG-3’ | Rat |
| **inhibitor N.C** | 5’-CAGUACUUUUGUGUAGUACAA-3’ | Rat |
| **mimics** |  |  |
| miR-145-5p | 5’-GUCCAGUUUUCCCAGGAAUCCCU-3’ | Rat |
| miR-23b-3p | 5’-AUCACAUUGCCAGGGAUUACC-3’ | Rat |
| miR-92a-3p | 5’-UAUUGCACUUGUCCCGGCCUG-3’ | Rat |
| miR-27a-3p | 5’-UUCACAGUGGCUAAGUUCCGC-3’ | Rat |
| miR-30a-5p | 5’-UGUAAACAUCCUCGACUGGAAG-3’ | Rat |
| **inhibitor** | | |
| miR-145-5p | 5’-AGGGAUUCCUGGGAAAACUGGAC-3’ | Rat |
| miR-23b-3p | 5’-GGUAAUCCCUGGCAAUGUGAU-3’ | Rat |
| miR-92a-3p | 5’-CAGGCCGGGACAAGUGCAAUA-3’ | Rat |
| miR-27a-3p | 5’-GCGGAACUUAGCCACUGUGAA-3’ | Rat |
| miR-30a-5p | 5’-CUUCCAGUCGAGGAUGUUUACA-3’ | Rat |

**Supplementary Table 2.** List of upregulated (fold change ≥ 1.5; p<0.05) and downregulated (fold change ≤ 0.7; p<0.05) miRNAs in chondrocytes treated with TNF-α (24 h, 10 ng/mL) compared with the untreated chondrocytes

| **Total：33** | **Fold Change**  (TNF-α/Ctrl) | **P-value** |
| --- | --- | --- |
| **Down-regulated (18)** | | |
| miR-1273c | 0.6190 | 0.02219 |
| miR-23b-3p | 0.2664 | 0.00234 |
| miR-9500 | 0.5767 | 0.03091 |
| miR-101a-3p | 0.6306 | 0.02517 |
| miR-513a-3p | 0.6205 | 0.02325 |
| miR-205-5p | 0.5104 | 0.01359 |
| miR-145-5p | 0.3426 | 0.01786 |
| miR-3118 | 0.5968 | 0.04341 |
| miR-134-5p | 0.5612 | 0.04523 |
| miR-92a-3p | 0.5821 | 0.04970 |
| miR-8073 | 0.6507 | 0.03477 |
| miR-3925-3p | 0.4028 | 0.00646 |
| miR-4795-5p | 0.5791 | 0.01268 |
| miR-30a-5p | 0.4370 | 0.03614 |
| miR-27a-3p | 0.5194 | 0.00319 |
| miR-127-5p | 0.6460 | 0.00327 |
| miR-6794-5p | 0.6170 | 0.00596 |
| miR-29b-3p | 0.6449 | 0.01578 |
| **Up-regulated (15)** | | |
| miR-4456 | 1.7807 | 0.04274 |
| miR-2682-3p | 2.1691 | 0.01698 |
| miR-34c-3p | 1.5673 | 0.03014 |
| miR-675-3p | 1.9461 | 0.03223 |
| miR-4448 | 1.7674 | 0.00932 |
| miR-302c-3p | 1.6370 | 0.04016 |
| miR-140-5p | 1.8483 | 0.04761 |
| miR-125b | 1.5228 | 0.02061 |
| miR-1911-5p | 1.5958 | 0.02126 |
| miR-7107-3p | 1.7000 | 0.01656 |
| miR-6879-3p | 1.5981 | 0.02523 |
| miR-130b-5p | 1.6097 | 0.03045 |
| miR-374b-5p | 1.6236 | 0.02125 |
| miR-18a-5p | 1.6042 | 0.00547 |
| miR-939-3p | 1.7699 | 0.03801 |

**Supplementary Table 3.** siRNAs

| **Gene (rat)** | **Sequence** |
| --- | --- |
| ***MKK4*** |  |
| siRNA#1 | 5ʹ-GGCAGAUAAUGGCAGUUAATT-3ʹ (sense)  5ʹ-UUAACUGCCAUUAUCUGCCTT-3ʹ (antisense) |
| siRNA#2 | 5ʹ-CUGGACAGGAGUGGAAAUATT-3ʹ (sense)  5ʹ-UAUUUCCACUCCUGUCCAGTT-3ʹ (antisense) |
| siRNA#3 | 5ʹ-GCUGAGUAAUUCCGAAGAATT-3ʹ (sense)  5ʹ-UUCUUCGGAAUUACUCAGCTT-3ʹ (antisense) |
| ***HGK*** |  |
| siRNA#1 | 5ʹ-GACGGAGAAAUACAUUCAUTT-3ʹ (sense)  5ʹ-AUGAAUGUAUUUCUCCGUCTT-3ʹ (antisense) |
| siRNA#2 | 5ʹ-GGUGCGUGCACUAUAAAGUTT-3ʹ (sense)  5ʹ-ACUUUAUAGUGCACGCACCTT-3ʹ (antisense) |
| siRNA#3 | 5ʹ-GCUCAGUCUAUGACAUUUATT-3ʹ (sense)  5ʹ-UAAAUGUCAUAGACUGAGCTT-3ʹ (antisense) |
| ***p65*** | 5ʹ-GUGGGCCUUAAUAGCCAUATT-3ʹ (sense)  5ʹ-UAUGGCUAUUAAGGCCCACTT-3ʹ (antisense) |
| ***Traf2*** | 5ʹ-GGAGAAGCUUCAGGACCAUTT-3ʹ (sense)  5ʹ-AUGGUCCUGAAGCUUCUCCTT-3ʹ (antisense) |
| ***Tradd*** | 5ʹ-CCAAGAAGAAAGUGGCAAUTT-3ʹ (sense)  5ʹ-AUUGCCACUUUCUUCUUGGTT-3ʹ (antisense) |

**Supplementary Table 4.** PCR primers

| **Gene (rat)** | **Sense primer (5'->3')** | **Antisense primer (5'->3')** |
| --- | --- | --- |
| *Sox-9* | CAGAGAACGCACATCAAGACG | GTAGGTGAAGGTGGAGTAGAGCC |
| *Col2a1* | CCTGAAACTCTGCCACCCAG | GTTCTTCCGAGGCACAGTCG |
| *Aggrecan* | TCATCCCGCTACGACGC | TCACCACCCACTCCGAAGA |
| *MMP-3* | TCTGGGCCTCCCTGAAAC | TGCCAATGCCTGGAAAGT |
| *MMP-13* | AAAGAACATGGTGACTTCTACC | ACTGGATTCCTTGAACGTC |
| *Adamts-4* | CCCGGAATGGTGGAAAGTATT | TCTTCACGGAAGGTCAATGCT |
| *Adamts-5* | CACGACCCTCAAGAACTTTTGC | TCACATGAATGATGCCCACATAA |
| *MKK4* | AATCGACAGCACGGTTTACTC | TGAAATCCCAGTGTTGTTCAGG |
| *HGK* | TACTCGGAAAGGCTCAGTGG | CATAAGGCAGCACACAGGAT |
| *TNF-α* | GAAACACACGAGACGCTGAA | ATCCACTCAGGCATCGACAT |
| *IL­1β* | TCTCACAGCAGCATCTCGAC | AAAGAAGGTGCTTGGGTCCT |
| *IL-6* | AGTTGCCTTCTTGGGACTGA | ACTGGTCTGTTGTGGGTGGT |
| *IL-8* | TTTTGCCAAGGAGTGCTAAAGA | AACCCTCTGCACCCAGTTTTC |
| *IL-17A* | GCGTCCTAAACAGAGACCTGA | AGGGTGGAAGGCAGACAAT |
| *IL-22* | CTGCTTCTCGTTGCTCTGTG | CATAAAGGTGCGGTTGACG |
| *VEGF* | TTGAGACCCTGGTGGACATCT | CTCCTATGTGCTGGCTTTGG |
| *p65* | TAGCCATAGTTGCGGTCCTT | CGTTCTTTCCTCCCTTTTCC |
| *Traf2* | TCACACAGACAGGCAGGAAG | CCCCAGACAGAAAATACAGCA |
| *Tradd* | GGCAATCTACAAGGCTCTGC | GAAACGCAACTGAACGATGA |
| *GAPDH* | GGCAAGTTCAACGGCACAG | CGCCAGTAGACTCCACGACA |
| *β-actin* | CTCTTCCAGCCTTCCTTCCT | TCATCGTACTCCTGCTTGCT |
| *U6* | CTCGCTTCGGCAGCACA | AACGCTTCACGAATTTGCGT |
| *miR-145*  (ChIP) | CACCTTGTCCTCACGGTCCAGTTTTCC | AGCCATGACCTCAAGAACAGTATTTC |
| *MMP-3*  (ChIP) | CTGAATTCTGTCTCCTAAGGATTGCCACC | CAGGTCTATAATTTTTTATTCCTTAGATG |
| *MMP-13*  (ChIP) | GCCAGATGAGTTTTGATATTCCCCC | CCACTTTGGGGTGTGTGTTAACTTCCTAG |
| *Adamts-5*  (ChIP) | CTGACAGTGAGTGGCTGACTTATAAG | GTGCCCACAATCTGTCACTTATCCC |

**Supplementary Figures:**

**
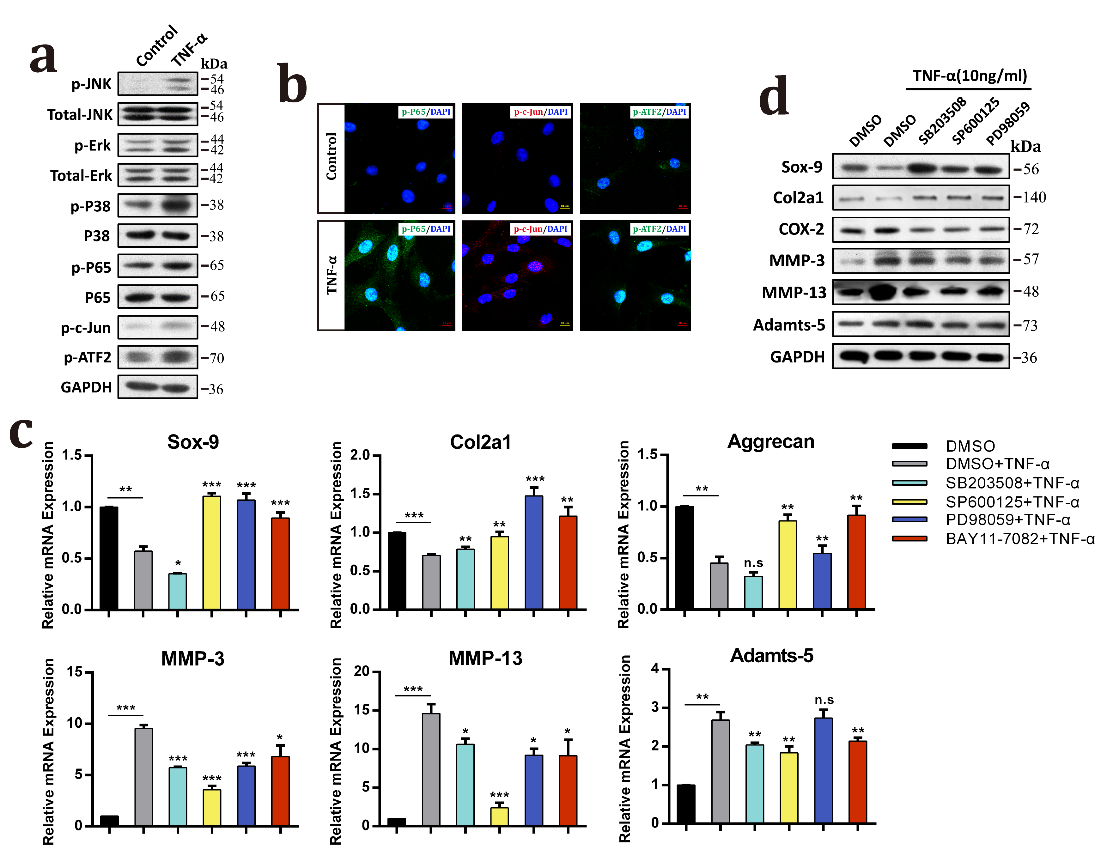
**

**Supplementary Figure 1. Blocking NF-κB and MAPK inhibits TNF-α-induced expression of matrix-degrading enzymes. (a)** Immunoblotting of the main molecules involved in the NF-κB and MAPK signaling pathways in TNF-α-stimulated chondrocytes. **(b)** Nuclear import of p-p65, p-c-Jun, and p-ATF2 induced by TNF-α. Scale bar: 10 μm. **(c)** The mRNA and **(d)** protein levels of catabolic and anabolic factors and pretreated with MAPK inhibitors (SB203508, SP600125, or PD98059, 10 μM) or NF-κB inhibitor (BAY11-7082, 5 μM) for 2 h and then cultured with or without TNF-α for 12 h. Data represent the mean±SEM of at least n=4 independent experiments. *p<0.05, **p<0.01, ***p<0.001, n.s=not significant.

**
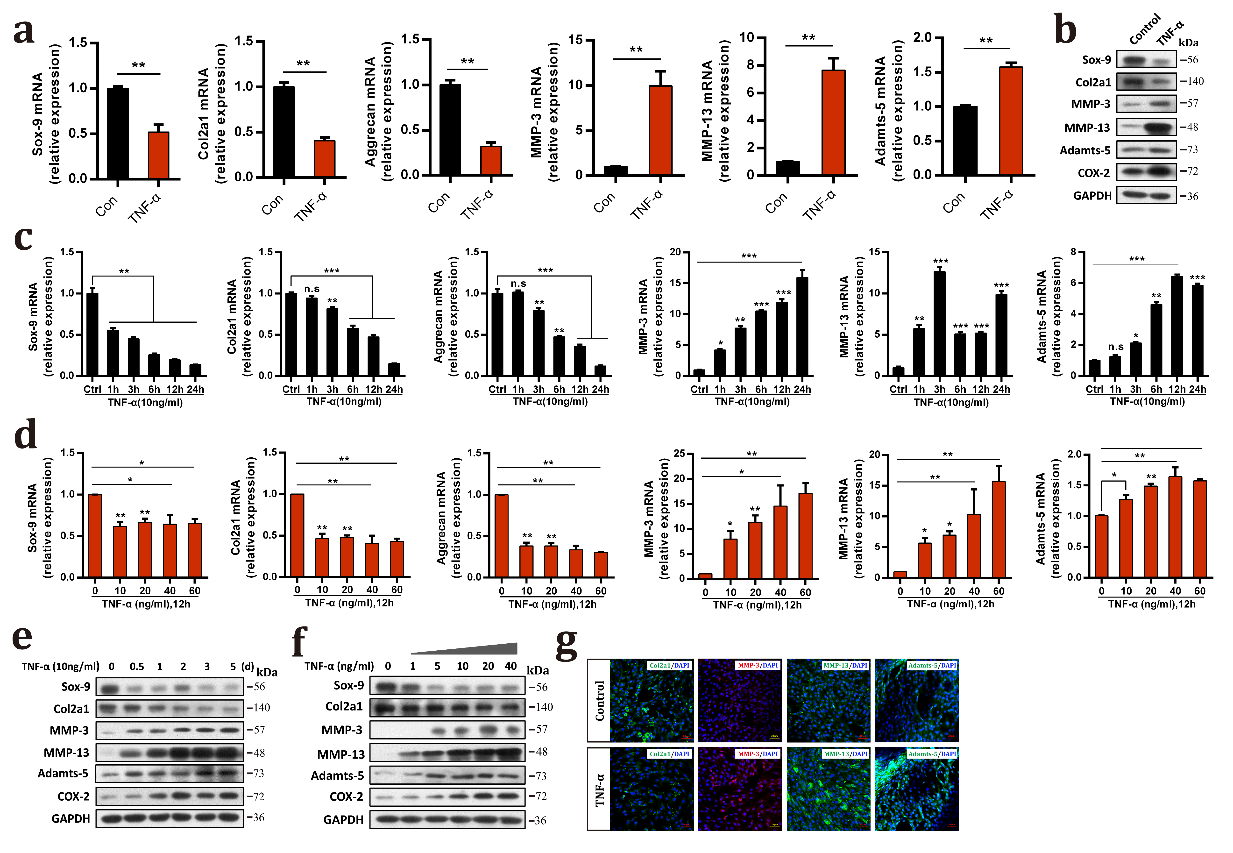
**

**Supplementary Figure 2. TNF-α promotes the catabolism and inhibits the anabolism of chondrocytes in time- and dose-dependent manner. (a)** The mRNA and **(b)** protein levels of catabolic and anabolic factors in chondrocytes stimulated with TNF-α (24 h,10 ng/mL). **(c**, **d**, **e** and **f)** Chondrocytes were stimulated with TNF-α (10 ng/mL) for different time periods or at different doses for 12 h. The mRNA levels of catabolic and anabolic factors were measured by qRT-PCR. Protein levels were analyzed by immunoblotting. **(g)** Immunofluorescence results of Col2a1, MMP-3, MMP-13, and Adamts-5. Scale bar: 20 μm. Data represent the mean±SEM of at least n=4 independent experiments. *p<0.05, **p<0.01, ***p<0.001, n.s=not significant.

**
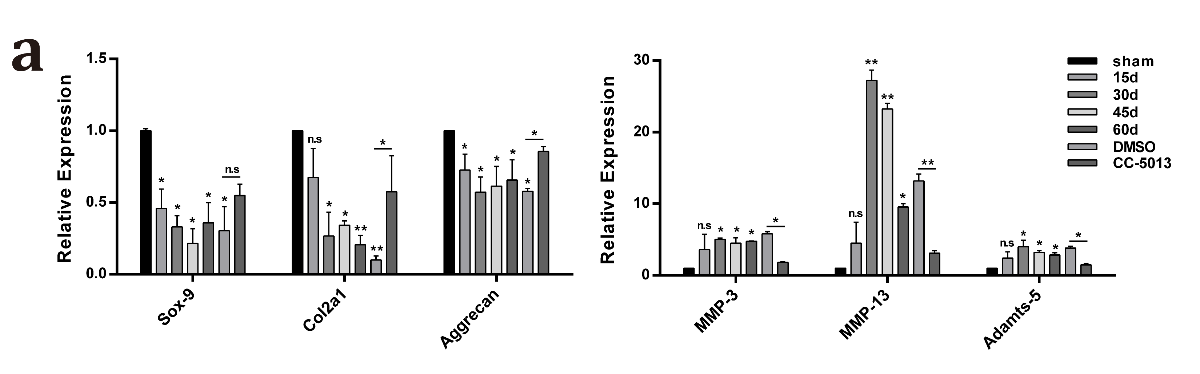
**

**Supplementary Figure 3. Pharmacological inhibition of TNF-α reduces the expression of matrix-degrading enzymes in experimental OA. (a)** The mRNA levels of Sox-9, Col2a1, Aggrecan, MMP-3, MMP-13, and Adamts-5 in rat cartilage obtained from the sham group (n=6), DMM groups (n=6), DMSO group (n=6) and CC-5013 group (n=6). Data represent the mean±SEM of at least n=4 independent experiments. *p<0.05, **p<0.01, ***p<0.001, n.s=not significant.

**
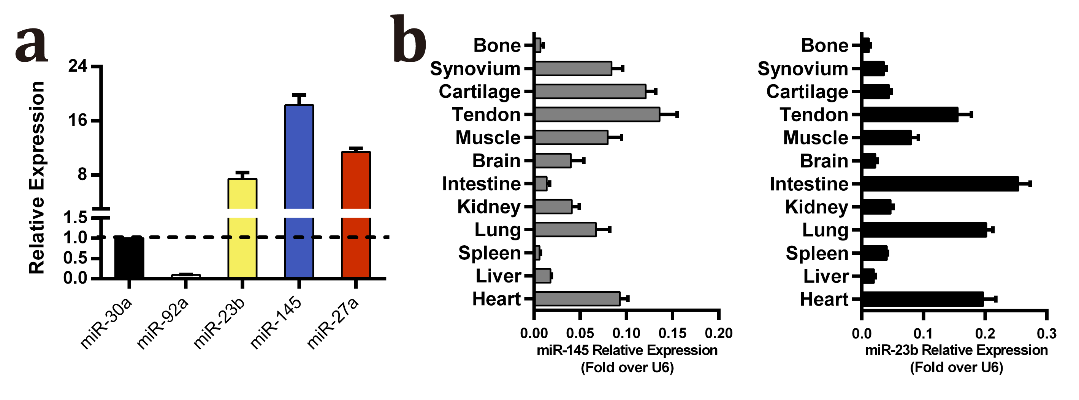
**

**Supplementary Figure 4. The expression profiles of miR-145 and miR-23b in chondrocytes and cartilage. (a)** Expression levels of the five miRNAs in rat primary chondrocytes. **(b)** Expression levels of miR-145 and miR-23b in different tissues from rats. Data represent the mean±SEM of at least n=4 independent experiments.


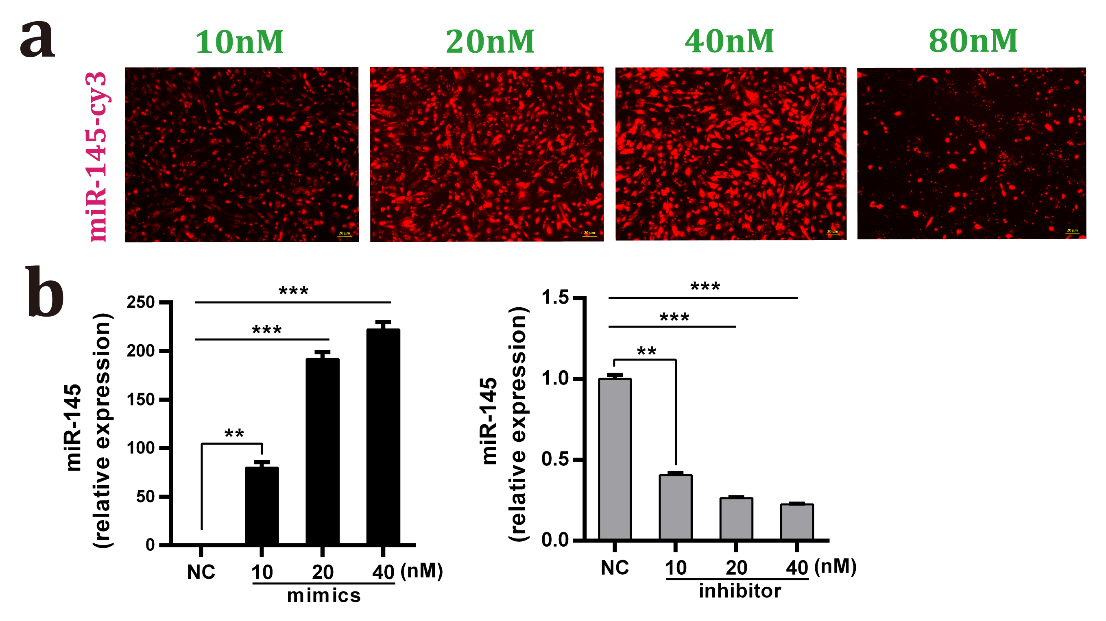


**Supplementary Figure 5. The transfection efficiency of miR-145 mimics and inhibitor. (a)** Chondrocytes were transfected with 20–25 nt oligonucleotides labeled by cy3 at different doses (10, 20, 40 nM, and 80 nM); the transfection efficiency was analyzed by immunofluorescence. Scale bar: 20 μm. **(b)** Expression level of miR-145 in chondrocytes transfected with miR-145 mimics, inhibitor, or their negative controls at different doses. Data represent the mean±SEM of at least n=4 independent experiments. *p<0.05, **p<0.01, ***p<0.001, n.s=not significant.


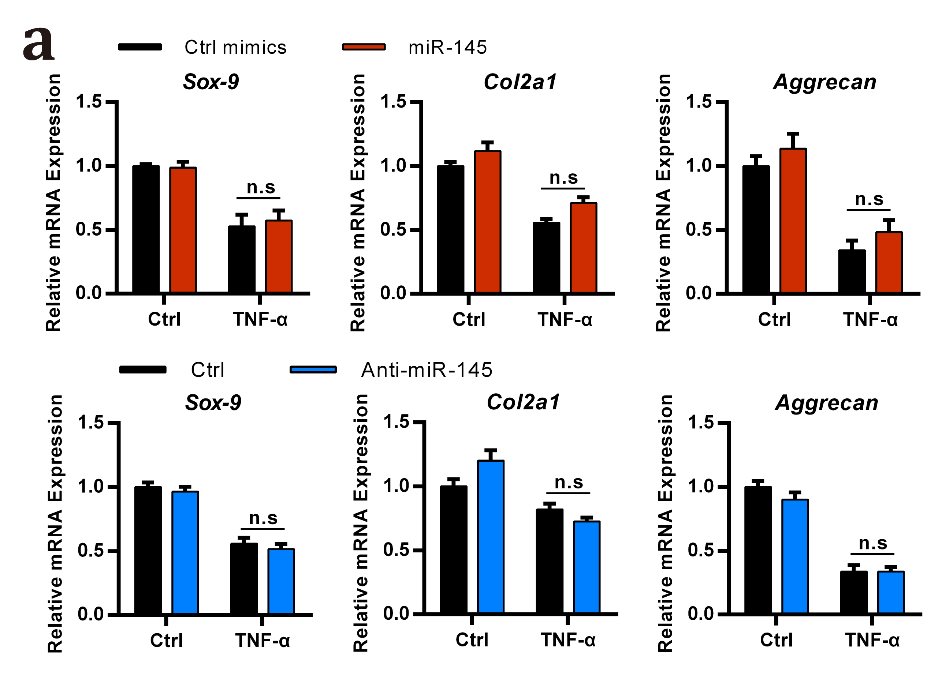


**Supplementary Figure 6. MiR-145 exerts no effect on anabolic factors of chondrocytes treated with TNF-α. (a)** Chondrocytes were transfected with miR-145 mimics, inhibitor or their negative controls. At 24 h after transfection, the cells were cultured with or without TNF-α. The mRNA levels of Sox-9, Col2a1, and Aggrecan were evaluated by qRT-PCR. Data represent the mean±SEM of at least n=4 independent experiments. n.s=not significant.


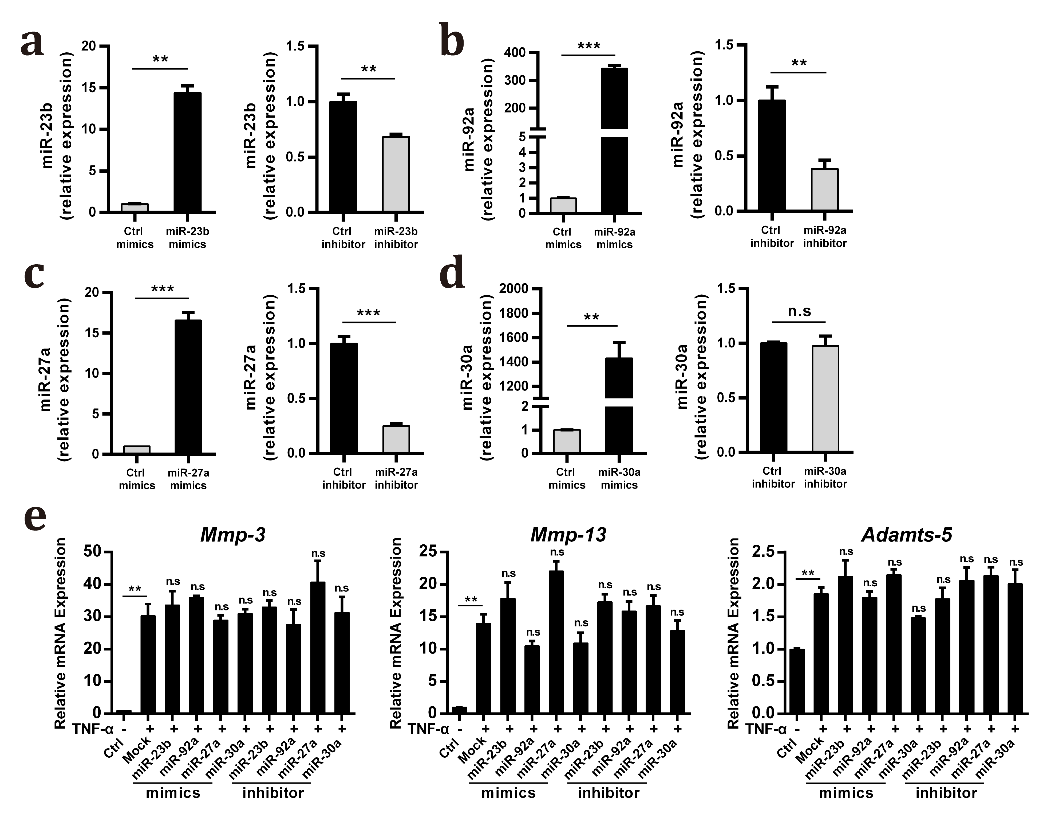


**Supplementary Figure 7. MicroRNAs not involved in TNF-α-triggered cartilage matrix degradation in chondrocytes. (a**, **b**, **c** and **d)** Mimics or inhibitor of the four microRNAs filtrated from the result of micro-array were transfected into chondrocytes at a final concentration of 40 nM. After 24 h, expression levels of miR-23b **(a)**, miR-92a **(b)**, miR-27a **(c)**, and miR-30a **(d)** were measured by qRT-PCR. **(e)** Chondrocytes were transfected with mimics, inhibitors, or negative controls of the miRNAs described above. At 24 h after transfection, the cells were cultured with or without TNF-α. The mRNA levels of MMP-3, MMP-13, and Adamts-5 were evaluated by qRT-PCR. Data represent the mean±SEM of at least n=4 independent experiments. *p<0.05, **p<0.01, ***p<0.001, n.s=not significant.


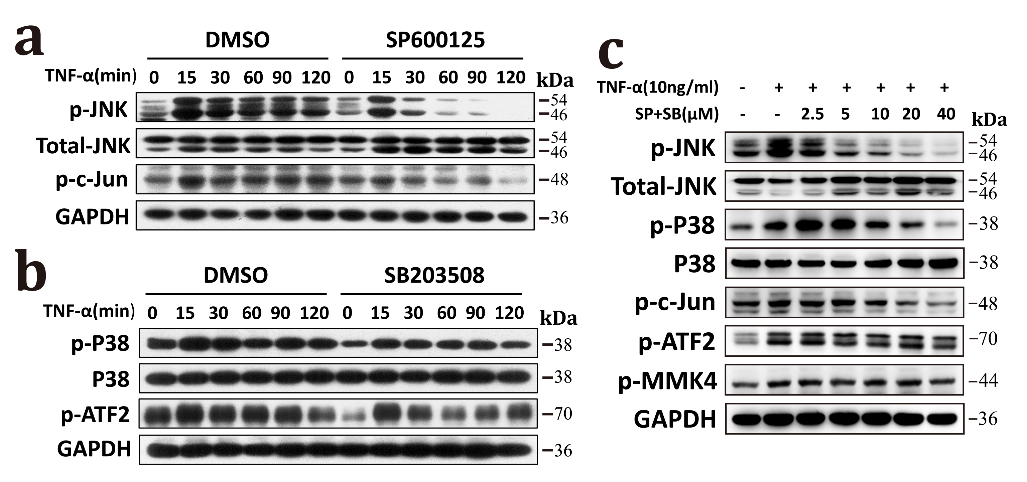


**Supplementary Figure 8. Inhibition of JNK and p38 signaling pathways with SP600125 and SB203508. (a)** Immunoblotting of p-JNK, JNK, and p-c-Jun in chondrocytes pretreated with SP600125 for 2 h and then stimulated with TNF-α for different time periods. **(b)** Immunoblotting of p-P38, P38, and p-ATF2 in chondrocytes pretreated with SB203508 for 2 h and then stimulated with TNF-α for different time periods. **(c)** The protein levels of p-MKK4, p-JNK, JNK, p-c-Jun, p-P38, P38, and p-ATF2 in chondrocytes pretreated with SP600125 in combination with SB203508 at different doses for 2 h and then stimulated with or without TNF-α for 15 min.

**
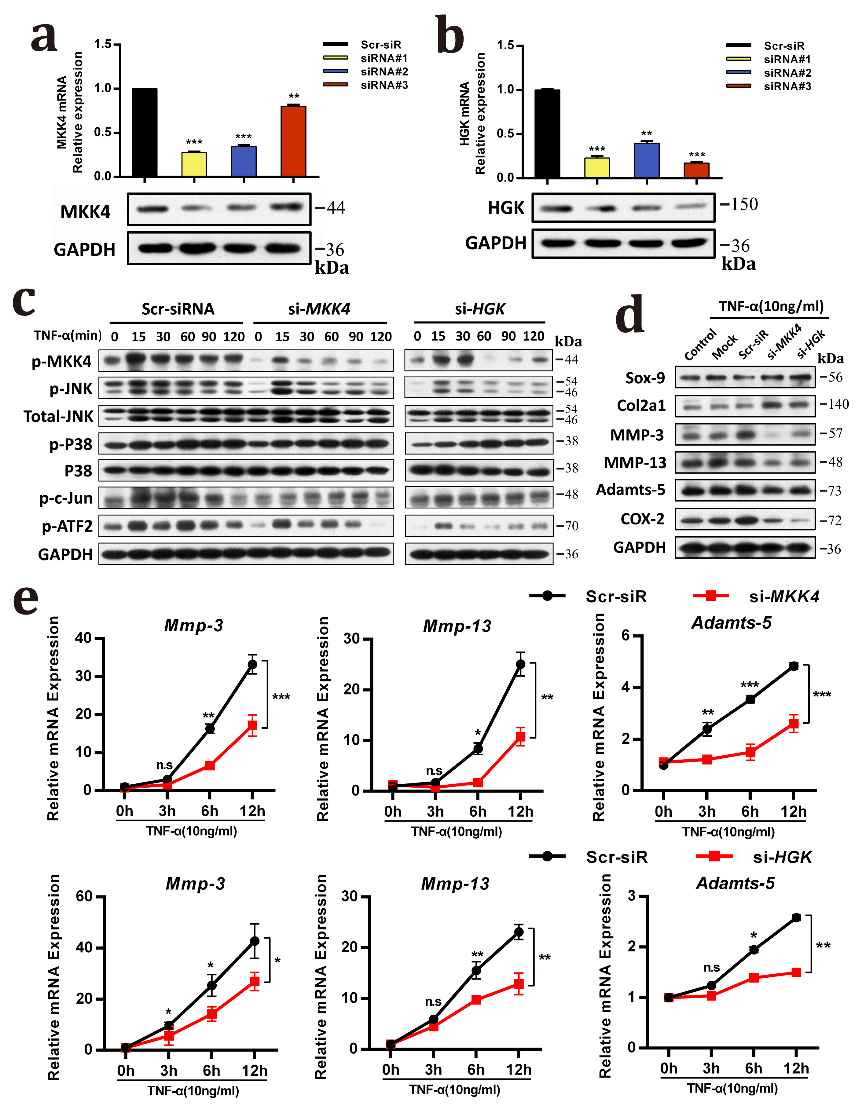
**

**Supplementary Figure 9. Knockdown of MKK4 or HGK inhibits TNF-α-induced matrix-degrading enzymes in chondrocytes. (a)** Chondrocytes were transfected with MKK4 siRNA (#1, #2, #3) or Scr-siR at a final concentration of 40 nM. At 48 h after transfection, the mRNA and protein levels of MKK4 were evaluated. **(b)** The mRNA and protein levels of HGK in chondrocytes transfected with HGK siRNA (#1,#2,#3) or Scr-siR. **(c)** Immunoblotting of p-MKK4, p-JNK, JNK, p-c-Jun, p-P38, P38, and p-ATF2 in chondrocytes transfected with MKK4 siRNA (#1), HGK siRNA (#3), or Scr-siR and then stimulated with TNF-α for different time periods as indicated. **(d)** The protein levels of catabolic and anabolic factors in chondrocytes transfected as described above and then stimulated with TNF-α for 48 h. **(e)** The mRNA levels of MMP-3, MMP-13, and Adamts-5 in chondrocytes transfected as described above and then stimulated with TNF-α for different time periods as indicated. Data represent the mean±SEM of at least n=4 independent experiments. *p<0.05, **p<0.01, ***p<0.001, n.s=not significant.

**
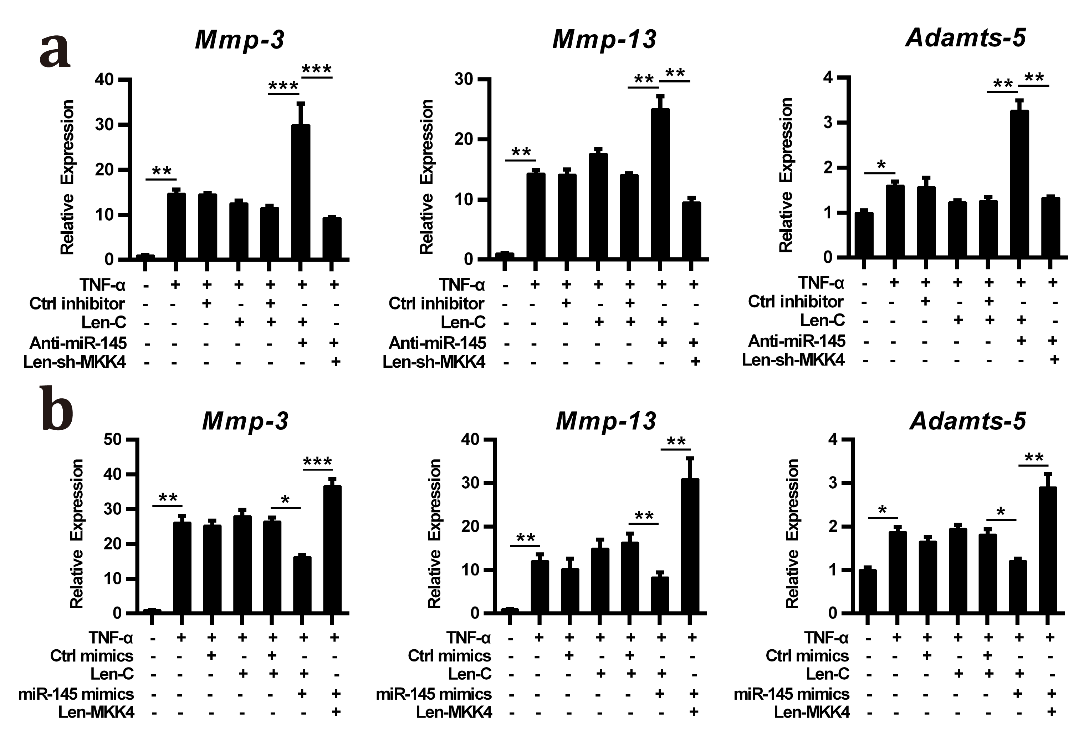
**

**Supplementary Figure 10. The inhibitory effect of miR-145 on TNF-α-induced upregulation of matrix-degrading enzymes is mediated by MKK4. (a)** The mRNA levels of MMP-3, MMP-13, and Adamts-5 in chondrocytes transfected with miR-145 inhibitor alone or in combination infected with Len-sh-*MKK4* (100 MOI) and then treated with TNF-α. **(b)** The mRNA levels of MMP-3, MMP-13, and Adamts-5 in chondrocytes transfected with miR-145 mimics alone or in combination infected with Len-*MKK4* (100 MOI) and then treated with TNF-α. Data represent the mean±SEM of at least n=4 independent experiments. *p<0.05, **p<0.01, ***p<0.001, n.s=not significant.


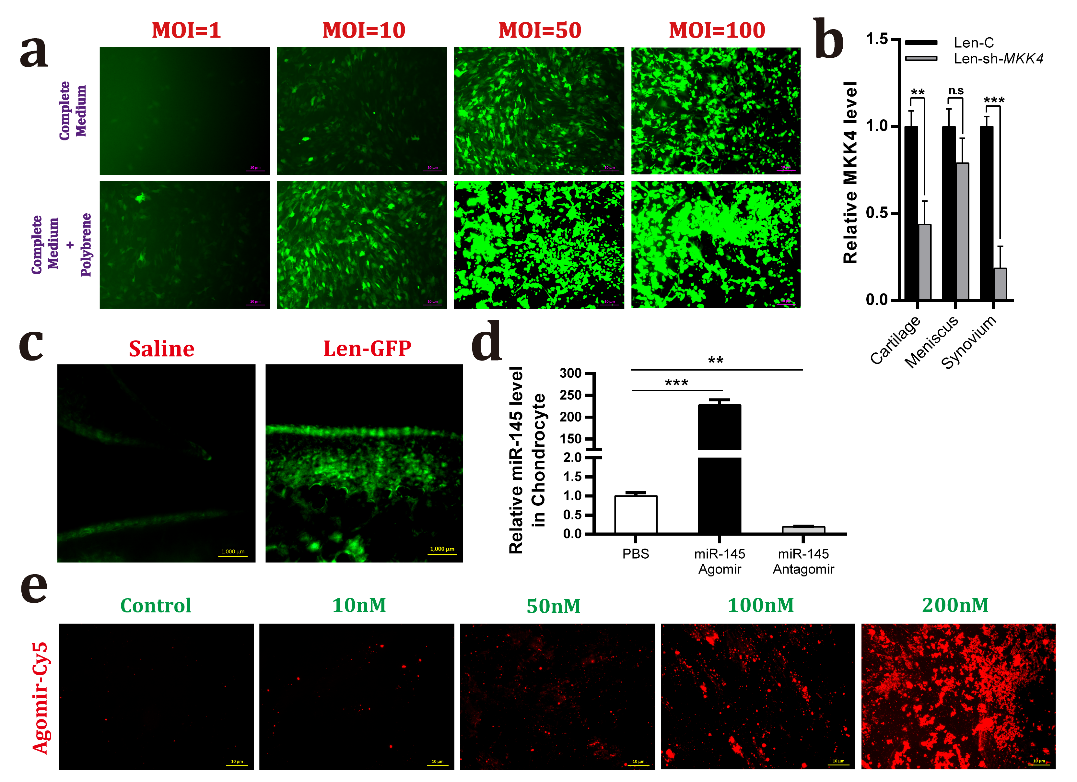


**Supplementary Figure 11. The delivery efficiency of lentivirus, agomir and antagomir into chondrocytes in vitro and in vivo. (a)** The infection efficiency of lentivirus containing GFP in chondrocytes at different MOIs with or without polybrene. Scale bar: 10 μm. **(b)** The mRNA level of MKK4 in the cartilage, meniscus, and synovium obtained from rats IA injected with Len-C and Len-sh-*MKK4* (n=6). **(c)** The infection efficiency of lentivirus containing GFP in the articular cartilage compared with the saline group (n=6). Scale bar: 1000 μm. **(d)** The mRNA level of MKK4 in chondrocytes treated with miR-145 agomir or antagomir without a transfection reagent. **(e)** The transfection efficiency of agomir labeled by cy5 in chondrocytes at different doses without a transfection reagent. Scale bar: 10 μm. Data represent the mean±SEM of at least n=4 independent experiments. *p<0.05, **p<0.01, ***p<0.001, n.s=not significant.
